# Supplementary material for: Enhancing emotion regulation with an in situ socially assistive robot among LGBTQ+ youth with self-harm ideation: protocol for a randomised controlled trial
Source: BMJ Open. 2024 Jan 9;14(1):e079801. doi: 10.1136/bmjopen-2023-079801 (PMC10806609; doi:10.1136/bmjopen-2023-079801)
Supplement: Supplementary data [file bmjopen-2023-079801supp001.pdf]

Supplementary material 1

Purrble logic model

Purrble operates on 3 levels:

1. Purrble directly provides in-the-moment soothing support in naturally occurring emotional moments when one would attempt to calm down. The physical and interactive design aims to tap into known regulatory factors using the extended Process Model of Emotion Regulation (Gross, 2015). This focuses on two separate stages of the ER process; i) the attentional deployment stage (Aldao et al., 2015; Sheppes & Gross, 2012; Sheppes et al., 2014; Farb et al., 2014) by shifting attention from the emotional situation towards interacting with the device, and ii) the response modulation stage (Beetz et al., 2012; Dore et al., 2017; Reeck et al., 2016; Coan et al., 2006; Crossman et al., 2018; Rabbit et al., 2015) by facilitating down-regulation through pleasant tactical interactions simulating the emotion regulatory effect of human-animal interactions.

2. Mechanisms of Purrble are designed to facilitate long-term engagement, by building on positive subjective experiences of in-the-moment soothing. As the device is framed as an anxious creature needing to be cared for, the key driver is that interactions are framed as helping regulate others' emotions (Dore et al., 2017; Cosley et al., 2010; Taylor, 2011), alongside facilitating a sense of relationship and responsibility for the well-being of the creature (Turkle, 2007; Hayashi & Kato, 2016; Donath, 2004; Lee et al., 2010).

3. Through repeated, soothing, and positive interactions with Purrble over time, it is anticipated that there will be a shift in ER practices and implicit beliefs about emotions (Ford & Gross, 2018), specifically the controllability of one's emotions (Schleider & Weisz, 2016).

Williams AJ, *et al. BMJ Open* 2024; 14:e079801. doi: 10.1136/bmjopen-2023-079801

Supplementary material 2

CONSENT FORM FOR PARTICIPANTS IN RESEARCH PROJECTS

Please complete this form after you have read the Information Sheet and/or listened to an explanation about the research

**Title of project:** An exploratory investigation of the acceptability and feasibility of using an in-situ socially assistive robot with at-risk young people.	
**Ethical review reference number: 34570**	**Version number: 3-23**

Please read and confirm your consent to taking part in this project by initialing all boxes, and signing (by typing your name) below:	Tick or initial
1. I confirm that I have read and understood the information sheet dated **3-23** for the above project. I have had the opportunity to consider the information and asked questions which have been answered to my satisfaction.	
2. I consent voluntarily to be a participant in this project and understand that I can refuse to take part and can withdraw from the project at any time, without having to give a reason, up to 2 weeks following my completion of the study.	
3. I consent to the processing of my personal information for the purposes explained to me in the Information Sheet. I understand that such information will be handled under the terms of UK data protection law, including the UK General Data Protection Regulation (UK GDPR) and the Data Protection Act 2018.	
4. I understand that my anonymised information may be subject to review by responsible individuals from the College for monitoring and audit purposes.	
5. I understand that confidentiality and anonymity will be maintained, and it will not be possible to identify me in any research outputs.	
6. I agree that the researcher/research team may use my data for future research and understand that any such use of identifiable data would be reviewed and approved by a research ethics committee. (In such cases, as with this project, data would not be identifiable in any report).	
7. I confirm that I am between 16-25 years old, who currently lives in the UK and identifies as any part of the LGBTQ+ umbrella.	
8. I understand that I need to provide the name and email address for my support contact (who will only be contacted to i) inform them that you are taking part in a mental health study, and ii) in the event that you can not be reached for a wellbeing check after 3 daily contact attempts via phone/email).	
9. I understand I will be randomised into either an intervention or control group, which impacts when I receive Purrble but irrespective I will be paid for all surveys completed across the 13 week period.	
10. I consent to completing three baseline surveys at the start of the study (weeks 1-3).	

11. I consent to be contacted via SMS to complete a weekly survey for 13 weeks (including the three baseline surveys).	
12. I consent to be invited to take part in an online interview to discuss my experience of the study.	
13. I consent to my interview being audio recorded, and shared with a third-party transcriber who will have signed a confidentiality agreement.	
14. I consent to be invited to take part in a co-designed workshop following the study.	
15. I confirm that I am happy to share my contact information as part of the study (name, email, phone number, home address)	
16. I understand that if I take part in the study, I will receive a Purrble through Royal Mail to my home address.	
17. I agree to take part in this project.	

Name:

Email address:

Your phone number:

Home address:

**Name of Participant**	**Date**	**Signature**
**Name of Researcher**	**Date**	**Signature**

If you have any questions or require further information, please contact

Researcher: A. Jess Williams

Email: amy_jess.williams@kcl.ac.uk

PI: Petr Slovak

Email: petr.slovak@kcl.ac.uk

3

Supplementary material 3

Full Measurement Details

Primary Outcome Measure

The primary outcome measure in this study is the Difficulties with Emotion Regulation Scale-8 (DERS8) [60]. This will be given to participants as part of the main assessment at all time-points. The DERS8 contains 8 items which represent affect, thoughts, and actions in response to situations which elicit negative emotions. A single total score is calculated from all items, with higher scores indicating more difficulties with emotion.

Mental Health Measures

Self-harmful thoughts and behaviours will be assessed using the Self-Harm Questionnaire (SHQ) [63]. At baseline, those who indicate experiences of self-harm behaviour will be invited to complete the second section about historical self-harm behaviour. At all other timepoints, only the three screening questions will be presented; these query the frequency of self-harmful thoughts, suicidal ideation, and self-harmful behaviour. The items are scored individually from "no thoughts/behaviour" to "yes, five or more times". The wording for these items will be adapted from "have you ever…" to "in the last week/month" (depending on assessment point).

Anxiety symptoms will be assessed using the Generalised Anxiety Disorder-7 (GAD-7) [64]. This is a seven-item instrument, used to identify or assess the severity of generalised anxiety disorder. Each item asks the individual to rate the severity of their symptoms over the time period. The total score is calculated by summing all items and ranges from 0-21. Higher scores indicate more severe levels of anxiety symptoms. The wording for these items will be adapted from "over the last two weeks" to "in the last week/month" (depending on assessment point).

Depressive symptoms will be assessed using the Patient Health Questionnaire-9 (PHQ9) [65]. Across nine items, this instrument measures the severity of depressive symptoms, the total score being calculated by summing all items with responses ranging from 0-27. Higher scores indicate greater depressive severity. Again, the wording for these items will be adapted from "over the last two weeks" to "in the last week/month" (depending on assessment point).

Proximal & Mechanistic Measures

All proximal and mechanistic measures will only be asked at three timepoints (T0, T5, T10), this is to reduce participant burden and encourage engagement, while also obtaining exploratory data to understand the impact of access to Purrble in greater detail. All additional measures total to 18 extra items, adding ~7 minutes to the assessment.

The Process Model of Emotion Regulation Questionnaire [66] is a 45-item measure which considers 10 ER strategies across the five stages of the Process Model of ER, and particularly how these strategies are used to decrease negative emotions. We will include 2 subscales

focusing on attention deployment (focusing elsewhere (4 items) and cognitive distraction (5 items)). Each subscale is scored by taking the average of item-level responses.

Hopefulness will be assessed using the State Hope Scale [67]. This is a six-item instrument concerning ongoing goal-directed thinking (agency and pathways). The total score (6-48) is calculated by summing responses, with higher scores relating to greater state hopefulness.

Loneliness is assessed using the 3-item UCLA loneliness scale for children [68] to consider participants' subjective loneliness. Items are summed to create a total score (3-12), whereby higher results indicate greater loneliness.

Engagement Measures

Engagement with Purrble will be assessed using two measures; a bespoke survey [59] and an adapted version of Twente Engagement with eHealth Technologies Scale (TWEETS) [69]. These will be deployed as part of the main assessment for participants allocated to the intervention group.

The bespoke survey [59] enquires about Purrble use and perceived usefulness over four items. These are analysed separately, with qualitative responses indicating contexts or situations where Purrble has been found helpful or unhelpful.

TWEETS [69] quantitatively measures intervention engagement across nine items. This is split into subsections considering behaviour, cognitive, and affective engagement. Total scores range from 0-36, with higher scores indicating greater engagement.

Williams AJ, *et al. BMJ Open* 2024; 14:e079801. doi: 10.1136/bmjopen-2023-079801

Supplementary material 4

SPIRIT

STANDARD PROTOCOL ITEMS: RECOMMENDATIONS FOR INTERVENTIONAL TRIALS

SPIRIT 2013 Checklist: Recommended items to address in a clinical trial protocol and related documents*

Section/item	Item No	Description	Page and line No
**Administrative information**			
Title	1	Descriptive title identifying the study design, population, interventions, and, if applicable, trial acronym	Page 1<br>1-4
Trial registration	2a	Trial identifier and registry name. If not yet registered, name of intended registry	Page 2<br>44
	2b	All items from the World Health Organization Trial Registration Data Set	n.a.
Protocol version	3	Date and version identifier	n.a.
Funding	4	Sources and types of financial, material, and other support	Page 13<br>399-401
Roles and responsibilities	5a	Names, affiliations, and roles of protocol contributors	Page 1<br>5-13<br><br>Page 13<br>391-396
	5b	Name and contact information for the trial sponsor	Page 13<br>400-401
	5c	Role of study sponsor and funders, if any, in study design; collection, management, analysis, and interpretation of data; writing of the report; and the decision to submit the report for publication, including whether they will have ultimate authority over any of these activities	Page 13<br>401-402

	5d	Composition, roles, and responsibilities of the coordinating centre, steering committee, endpoint adjudication committee, data management team, and other individuals or groups overseeing the trial, if applicable (see Item 21a for data monitoring committee)	n.a.
**Introduction**			
Background and rationale	6a	Description of research question and justification for undertaking the trial, including summary of relevant studies (published and unpublished) examining benefits and harms for each intervention	Page 2-4<br>55-149
	6b	Explanation for choice of comparators	Page 6<br>195-199<br><br>Page 12<br>360-362
Objectives	7	Specific objectives or hypotheses	Page 4<br>150-161
Trial design	8	Description of trial design including type of trial (eg, parallel group, crossover, factorial, single group), allocation ratio, and framework (eg, superiority, equivalence, noninferiority, exploratory)	Page 4-5<br>163-178
**Methods: Participants, interventions, and outcomes**			
Study setting	9	Description of study settings (eg, community clinic, academic hospital) and list of countries where data will be collected. Reference to where list of study sites can be obtained	Page 6-7<br>204-222
Eligibility criteria	10	Inclusion and exclusion criteria for participants. If applicable, eligibility criteria for study centres and individuals who will perform the interventions (eg, surgeons, psychotherapists)	Page 6<br>201-209
Interventions	11a	Interventions for each group with sufficient detail to allow replication, including how and when they will be administered	Page 5-6<br>179-199
	11b	Criteria for discontinuing or modifying allocated interventions for a given trial participant (eg, drug dose change in response to harms, participant request, or improving/worsening disease)	Page 7<br>234-235

Williams AJ, *et al. BMJ Open* 2024; 14:e079801. doi: 10.1136/bmjopen-2023-079801

	11c	Strategies to improve adherence to intervention protocols, and any procedures for monitoring adherence (eg, drug tablet return, laboratory tests)	Page 8<br>285-287
	11d	Relevant concomitant care and interventions that are permitted or prohibited during the trial	n.a.
Outcomes	12	Primary, secondary, and other outcomes, including the specific measurement variable (eg, systolic blood pressure), analysis metric (eg, change from baseline, final value, time to event), method of aggregation (eg, median, proportion), and time point for each outcome. Explanation of the clinical relevance of chosen efficacy and harm outcomes is strongly recommended	Page 8-10<br>268-292<br><br>SupMat3
Participant timeline	13	Time schedule of enrolment, interventions (including any run-ins and washouts), assessments, and visits for participants. A schematic diagram is highly recommended (see Figure)	Page 6-7<br>211-244
Sample size	14	Estimated number of participants needed to achieve study objectives and how it was determined, including clinical and statistical assumptions supporting any sample size calculations	Page 7-8<br>245-267
Recruitment	15	Strategies for achieving adequate participant enrolment to reach target sample size	Page 6-7<br>211-222
**Methods: Assignment of interventions (for controlled trials)**			
Allocation:			
Sequence generation	16a	Method of generating the allocation sequence (eg, computer-generated random numbers), and list of any factors for stratification. To reduce predictability of a random sequence, details of any planned restriction (eg, blocking) should be provided in a separate document that is unavailable to those who enrol participants or assign interventions	Page 7<br>224-228
Allocation concealment mechanism	16b	Mechanism of implementing the allocation sequence (eg, central telephone; sequentially numbered, opaque, sealed envelopes), describing any steps to conceal the sequence until interventions are assigned	Page 7<br>224-228
Implementation	16c	Who will generate the allocation sequence, who will enrol participants, and who will assign participants to interventions	Page 7<br>224-231

Blinding (masking)	17a	Who will be blinded after assignment to interventions (eg, trial participants, care providers, outcome assessors, data analysts), and how	Page 7<br>224-231
	17b	If blinded, circumstances under which unblinding is permissible, and procedure for revealing a participant's allocated intervention during the trial	n.a.
**Methods: Data collection, management, and analysis**			
Data collection methods	18a	Plans for assessment and collection of outcome, baseline, and other trial data, including any related processes to promote data quality (eg, duplicate measurements, training of assessors) and a description of study instruments (eg, questionnaires, laboratory tests) along with their reliability and validity, if known. Reference to where data collection forms can be found, if not in the protocol	Page 4-5<br>163-178<br><br>Page 7-10<br>245-292<br><br>SupMat3
	18b	Plans to promote participant retention and complete follow-up, including list of any outcome data to be collected for participants who discontinue or deviate from intervention protocols	n.a.
Data management	19	Plans for data entry, coding, security, and storage, including any related processes to promote data quality (eg, double data entry; range checks for data values). Reference to where details of data management procedures can be found, if not in the protocol	Digital Youth management agreement
Statistical methods	20a	Statistical methods for analysing primary and secondary outcomes. Reference to where other details of the statistical analysis plan can be found, if not in the protocol	Page 11-12<br>307-357
	20b	Methods for any additional analyses (eg, subgroup and adjusted analyses)	Page 11-12<br>327-336<br><br>352-357
	20c	Definition of analysis population relating to protocol non-adherence (eg, as randomised analysis), and any statistical methods to handle missing data (eg, multiple imputation)	n.a.
**Methods: Monitoring**			

Data monitoring	21a	Composition of data monitoring committee (DMC); summary of its role and reporting structure; statement of whether it is independent from the sponsor and competing interests; and reference to where further details about its charter can be found, if not in the protocol. Alternatively, an explanation of why a DMC is not needed	Page 13<br>400-403
	21b	Description of any interim analyses and stopping guidelines, including who will have access to these interim results and make the final decision to terminate the trial	n.a.
Harms	22	Plans for collecting, assessing, reporting, and managing solicited and spontaneously reported adverse events and other unintended effects of trial interventions or trial conduct	Page 7<br>237-244<br><br>SupMat5
Auditing	23	Frequency and procedures for auditing trial conduct, if any, and whether the process will be independent from investigators and the sponsor	n.a.
**Ethics and dissemination**			
Research ethics approval	24	Plans for seeking research ethics committee/institutional review board (REC/IRB) approval	Page 12-13<br>367-369
Protocol amendments	25	Plans for communicating important protocol modifications (eg, changes to eligibility criteria, outcomes, analyses) to relevant parties (eg, investigators, REC/IRBs, trial participants, trial registries, journals, regulators)	Page 13<br>375-381
Consent or assent	26a	Who will obtain informed consent or assent from potential trial participants or authorised surrogates, and how (see Item 32)	Page 7<br>224-225<br><br>Page 12-13<br>369-372
	26b	Additional consent provisions for collection and use of participant data and biological specimens in ancillary studies, if applicable	n.a.
Confidentiality	27	How personal information about potential and enrolled participants will be collected, shared, and maintained in order to protect confidentiality before, during, and after the trial	SupMat2
Declaration of interests	28	Financial and other competing interests for principal investigators for the overall trial and each study site	Page 13-14<br>404-410

Access to data	29	Statement of who will have access to the final trial dataset, and disclosure of contractual agreements that limit such access for investigators	Page 14 412-415
Ancillary and post-trial care	30	Provisions, if any, for ancillary and post-trial care, and for compensation to those who suffer harm from trial participation	n.a.
Dissemination policy	31a	Plans for investigators and sponsor to communicate trial results to participants, healthcare professionals, the public, and other relevant groups (eg, via publication, reporting in results databases, or other data sharing arrangements), including any publication restrictions	Page 13 375-381
	31b	Authorship eligibility guidelines and any intended use of professional writers	n.a.
	31c	Plans, if any, for granting public access to the full protocol, participant-level dataset, and statistical code	n.a.
**Appendices**			
Informed consent materials	32	Model consent form and other related documentation given to participants and authorised surrogates	SupMat2
Biological specimens	33	Plans for collection, laboratory evaluation, and storage of biological specimens for genetic or molecular analysis in the current trial and for future use in ancillary studies, if applicable	n.a.

*It is strongly recommended that this checklist be read in conjunction with the SPIRIT 2013 Explanation & Elaboration for important clarification on the items. Amendments to the protocol should be tracked and dated. The SPIRIT checklist is copyrighted by the SPIRIT Group under the Creative Commons “Attribution-NonCommercial-NoDerivs 3.0 Unported” license.

Supplementary material 5

Safeguarding Procedures

The safeguarding procedures will be presented to potential participants in the information sheet before they consent to the study. These were codesigned and agreed with Sprouting Minds, based on previous studies conducted with young people with self-harmful experiences [1-3]. To support young people's safety during the study, the following measures will be instituted:

1. Before starting the study, all participants are required to attend an individual study briefing. At this point, they are taken through the study outline, invited to ask questions, and asked to create a safety plan [4] with the support of a specially trained researcher. Safety planning is quick to complete, is tangible to participants and has been shown to be effective at reducing self-harmful thoughts and behaviours [5]. Participants keep this safety plan containing their individualised strategies for their personal use beyond the life of the study. During the wellbeing check, participants are asked to reflect on their safety plan if they feel distressed at any other point during the study. This follows safety protocols used in other studies by the researcher [1-3].
2. During the briefing, participants will need to nominate a support contact (parent, friend over 18 years, GP, etc), with the understanding that if they do not respond to reactive safeguarding (which can be triggered for each of the main assessments), this person will be contacted to ensure that the participant is safe.
    a. This nominated person will be contacted (via email) to inform them that the participant is taking part in a mental health study and that we will reach out if we are unable to contact the participant following indication that they are at increased risk.
3. All assessments will include signposting to additional supports (e.g., Young Minds, Samaritans, Papyrus) as well as encouraging participants to seek help from their GP if they are distressed.
4. All assessments will include a visual scale to rate mood pre- and post- completing the survey (1 - very distressed - 10 extremely happy). This is to examine whether the assessment process has an impact, positively or negatively, on the participant. Such assessments have been successfully used in prior self-harm research by the research team members as an indicator of assessment impact [36].
5. Participants in need of support will be identified by checking their responses to the main battery of assessments, within 24 hours. If participants respond that they have had experiences of self-harmful or suicidal thoughts and self-harm behaviour, as well as showing a that the assessment has had a negative impact, they receive a wellbeing call the following day between 1pm-4pm. This allows enough time for the researcher to identify participant risk and inform the PI.
    a. If the participant does not pick up the phone, they will be contacted via email, asking about their wellbeing, whether they wish to continue with the study, and to arrange a time convenient to complete a wellbeing check. During the wellbeing check, the researcher will be empathic to the difficulties of the participant. The participant will be asked whether they wish to continue the study and, if so, they

Williams AJ, *et al. BMJ Open* 2024; 14:e079801. doi: 10.1136/bmjopen-2023-079801

will update their safety plan with the researcher. If participants do not wish to continue the study, they will be allowed to withdraw with no consequences. They will be invited to interview to discuss their thoughts, opinions and experiences of the study. This would also ask about why they withdrew from the study.

b. If a participant still does not respond to the email, the same procedure will be attempted twice more. If there is still no contact from the participant by day 4, their support contact (nominated during briefing) will be contacted.

Alongside this, all researchers who undertake briefing (including safety planning) and wellbeing calls will be invited to take part in group supervision sessions once a week during data collection with leading authors. This will be a confidential, safe space to discuss researchers' wellbeing and mental health, with support for any difficulties which may surface.

References

1. Williams AJ, Arcelus J, Townsend E, Michail M. Feasibility and acceptability of experience sampling among LGBTQ+ young people with self-harmful thoughts and behaviours. Frontiers in psychiatry. 2022 Aug 17;13:916164. Doi.org/10.3389/fpsyt.2022.916164
2. Williams AJ, Arcelus J, Townsend E, Michail M. Understanding the processes underlying self-harm ideation and behaviors within LGBTQ+ young people: A qualitative study. Archives of suicide research. 2021 Apr 3;27(2):380-96. doi.org/10.1080/13811118.2021.2003273
3. Williams, AJ, Townsend, T, Naeche, N, Chapman-Nisar, A, Hollis, C, Slovak, P. Investigating the feasibility, acceptability and appropriation of a socially assistive robot among minority youth at-risk of self-harm. [in prep]
4. Stanley B, Brown GK. Safety planning intervention: a brief intervention to mitigate suicide risk. Cognitive and behavioral practice. 2012 May 1;19(2):256-64. doi.org/10.1016/j.cbpra.2011.01.001
5. Nuij C, van Ballegooijen W, De Beurs D, Juniar D, Erlangsen A, Portzky G, O'Connor RC, Smit JH, Kerkhof A, Riper H. Safety planning-type interventions for suicide prevention: meta-analysis. The British Journal of Psychiatry. 2021 Aug;219(2):419-26. doi.org/10.1192/bjp.2021.50
