# Supplementary material for: Enhancing emotion regulation with an in situ socially assistive robot among LGBTQ+ youth with self-harm ideation: protocol for a randomised controlled trial
Source: BMJ Open. 2024 Jan 9;14(1):e079801. doi: 10.1136/bmjopen-2023-079801 (PMC10806609; doi:10.1136/bmjopen-2023-079801)
Supplement: Author's manuscript [file bmjopen-2023-079801.draft_revisions.pdf]

BMJ Open is committed to open peer review. As part of this commitment we make the peer review history of every article we publish publicly available.

When an article is published we post the peer reviewers' comments and the authors' responses online. We also post the versions of the paper that were used during peer review. These are the versions that the peer review comments apply to.

The versions of the paper that follow are the versions that were submitted during the peer review process. They are not the versions of record or the final published versions. They should not be cited or distributed as the published version of this manuscript.

BMJ Open is an open access journal and the full, final, typeset and author-corrected version of record of the manuscript is available on our site with no access controls, subscription charges or pay-per-view fees (http://bmjopen.bmj.com).

If you have any questions on BMJ Open's open peer review process please email info.bmjopen@bmj.com

Enhancing emotion regulation with an in-situ socially assistive robot among LGBTQ+ youth with self-harm ideation: Protocol for a randomised controlled trial

Journal:	*BMJ Open*
Manuscript ID	bmjopen-2023-079801
Article Type:	Protocol
Date Submitted by the Author:	13-Sep-2023
Complete List of Authors:	Williams, A. Jess; King's College London, Informatics; University of Nottingham, Institute of Mental Health<br>Borschmann, Rohan; Murdoch Childrens Research Institute; University of Oxford<br>Tench, Christopher; University of Nottingham, Division of Clinical Neuroscience<br>Cleare, Seonaid; University of Glasgow<br>Gross, James; Stanford University<br>Hollis, Chris; University of Nottingham, Division of Psychiatry<br>Chapman-Nisar, Amelia; University of Nottingham<br>Naeche, Nkem; University of Nottingham<br>Townsend, Ellen; University of Nottingham, School of Psychology<br>Slovak, Petr; King's College London; University of Oxford
Keywords:	Suicide & self-harm < PSYCHIATRY, MENTAL HEALTH, Adolescents < Adolescent, Sexual and Gender Minorities, Protocols & guidelines < HEALTH SERVICES ADMINISTRATION & MANAGEMENT, Randomized Controlled Trial

Enhancing emotion regulation with an in-situ socially assistive robot among LGBTQ+ youth with self-harm ideation: Protocol for a randomised controlled trial

A. Jess Williams*, Rohan Borschmann, Christopher Tench, Seonaid Cleare, James J Gross, Chris Hollis, Amelia Chapman-Nisar, Nkem Naeche, Ellen Townsend, Petr Slovak

*Corresponding author: amy_jess.williams@kcl.ac.uk
Informatics,

Bush House

Strand Campus

King's College London

Abstract:

Introduction: Purrble, a socially assistive robot, was co-designed with children to support in-situ emotion regulation. Preliminary evidence has found that LGBTQ+ youth are receptive to Purrble and find it to be an acceptable intervention to assist with emotion dysregulation and their experiences of self-harm. The present study is designed to evaluate the impact of access to Purrble among LGBTQ+ youth who have self-harmful thoughts, when compared to waitlist controls.

Methods and analysis: The study is a single-blind, randomised control trial comparing access to the Purrble device with waitlist control. A total of 168 LGBTQ+ youth aged 16-25 years with current self-harmful ideation will be recruited, all based within the UK.

The primary outcome is emotion dysregulation (Difficulties with Emotion Regulation Scale-8) measured weekly across a 13-week period, including 3 pre-deployment timepoints. Secondary outcomes include self-harm (Self-Harm Questionnaire), anxiety (Generalised Anxiety Disorder-7), and depression (Patient Health Questionnaire-9). We will conduct analyses using linear mixed models to assess primary and secondary hypotheses.

For peer review only

After the study has ended, a subset of participants will be invited to participate in semi-structured interviews to explore engagement and appropriation of Purrble, considering the young people's own views of Purrble as an intervention device.

Ethics and dissemination: Ethical approval was received from King's College London (RESCM-22/23-34570). Findings will be disseminated in peer review open-access journals and at academic conferences.

Trial registration number: Clinical trial gov: NCT06025942

Strengths and limitations of this study

- This is the first randomised controlled trial to explore the impact of access of Purrble, a socially assistive robot, compared to waitlist control on emotion regulation difficulties within LGBTQ+ youth who have current experiences of self-harmful ideation.
- Purrble was co-designed with youth to support emotion regulation in-situ.
- The study was co-designed with young people who have experience of mental health difficulties (Sprouting Minds), including a detailed safeguarding procedure.
- Participants will not be blinded to participant group due to the nature of the intervention.

Introduction

Self-harm, defined as the intentional poisoning or injury of self, irrespective of intention [1], is a key health concern among sexual orientation and/or gender identity minorities, LGBTQ+ populations [2]. Internationally, young LGBTQ+ people report higher prevalence of self-harmful thoughts and behaviours, anxiety, depression, and substance misuse when compared to their cisgender, heterosexual peers [3-8] It is well documented that those with a history of self-harm are at greater risk of suicide [9], and recent evidence indicates that LGBTQ+ youth are 3-6 times more likely attempt suicide than their cisgender, heterosexual counterparts [10-11]. Despite the considerable risk of self-harm and associated adverse outcomes [12-13], there is a lack of evidence-based interventions to support LGBTQ+ youth struggling with self-harmful thoughts and behaviours.

Youth who self-harm often do not seek professional help [14-15], and those that do find services (e.g., Child and Adolescent Mental Health Services, Accident and Emergency or social services) to be less helpful sources of support [16] or rarely re-attend services [17].

For peer review only - http://bmjopen.bmj.com/site/about/guidelines.xhtml

Factors relating to this can include negative attitudes and behaviours/treatment from health care staff (e.g., withdrawing pain-reducing medication for wound treatment [18]), concerns about confidentiality [19], or perceived stigma surrounding self-harm [20-21]. Among LGBTQ+ people, help-seeking is even more complex, with one in seven avoiding services due to fears of discrimination on the basis of their sexual orientation or gender identity [22]. Therefore, community-based interventions may be more appropriate to support LGBTQ+ youth engaging in self-harm.

As LGBTQ+ youth are frequent users of digital technologies [23-26], there is an opportunity for digital interventions to support those struggling with self-harm and other mental health difficulties. Evidence suggests that digital interventions support youth to bypass various barriers to help-seeking, such as lack of accessibility, anticipated stigma, inadequate resources, and the desire to be self-reliant [27], which are compounded by unique challenges facing LGBTQ+ youth (e.g. concerns about experiencing stigma or discrimination as a minority [28]).

For peer review only

At present, the field concerning digital interventions among LGBTQ+ youth is small, yet those available are perceived as feasible, acceptable, and relatively effective [29]. However, most focus on physical health such as risk reduction or management of sexually transmitted illnesses (STIs; [29]), with few concerning mental wellbeing [30-33]. These mental health interventions are typically perceived positively by LGBTQ+ youth [30, 32-34], with mixed findings reported by the three considering the impact of the intervention on participants at this stage; 1) Rainbow SPARX [32]; 2) an online writing intervention [31]; and 3) QueerViBE [30]. In their pilot trial, Rainbow SPARX (a didactic PC game using Cognitive Behaviour Therapy principles) was associated with large reduction for depressive (d=1.01) and anxious (d=0.95) symptoms [32]. Whereas, QueerViBE (a series of brief, interactive videos designed for transgender and gender diverse youth) found a moderate decrease in psychological distress (d=0.63) when compared to the control group [30]. However, the expressive writing intervention demonstrated no difference in depressive symptoms in their randomised controlled trial [31]. Therefore, while limited, digital interventions are feasible, acceptable and potentially effective for improving mental health among LGBTQ+ youth. However, there are

For peer review only - http://bmjopen.bmj.com/site/about/guidelines.xhtml

currently no evidence-based digital interventions targeting LGBTQ+ youth who struggle with self-harm.

Whilst self-harm among LGBTQ+ youth can be associated with multiple risks, complex experiences, and unique stressors [35-37], a common issue is often emotional dysregulation [38-41]. Emotion regulation (ER) is a well-known transdiagnostic risk factor [42-44], which can be associated with higher risk of self-harm across ages, settings, and genders [45-46]. Typically, LGBTQ+ populations report greater difficulties with ER [40-41], which explains in part the association between LGBTQ+ identity and self-harm [40-41]. Examining how ER can be better supported in young LGBTQ+ people who self-harm through digital intervention may be a helpful preventative strategy to aid LGBTQ+ youth broadly.

To address this, a pilot study was conducted with LGBTQ+ youth who had recent experiences of self-harmful thoughts and/or behaviours using an in-situ, emotion regulation intervention tool, Purrble [47], designed to provide in-the-moment support see Intervention section for further details. Purrble was originally developed for children in moments of situated distress, but has been well-accepted across child and student populations delivering notable benefits for emotion regulation [47-50]. Among a small sample of 21 LGBTQ+ young people, Purrble was found to be a feasible and acceptable intervention with continued device engagement across a 2-week deployment [47]. Notably, access to Purrble was also associated with a reduction in anxiety symptoms and self-harmful thoughts. Qualitative data indicated that this was linked to Purrble supporting ER practices (e.g., grounding, soothing) to prevent young people acting on their self-harmful urges and, in some cases, preventing them from considering self-harm at all [47]. Based on these findings, and Purrble's original design to support in-situ, bottom-up ER [48-49], it appears that mental health outcomes such as anxiety and self-harm [47, 50] are guided by the proximal change in emotion regulation.

Although these early data are promising, there is a lack of robust quantitative data on the impact of the intervention in a wider sample of LGBTQ+ young people who have self-harmed. Evidence is therefore urgently needed to evaluate the efficacy of Purrble in a)

For peer review only - http://bmjopen.bmj.com/site/about/guidelines.xhtml

delivering measurable changes in ER when compared to a control group, and b) the extent to which this impacts the frequency of self-harmful thoughts and/or anxiety symptoms.

Study objectives

Primary Objective: The primary objective of this study is to evaluate the impact of having access to the Purrble intervention, compared with a waitlist control, on emotion regulation difficulties (DERS8) among LGBTQ+ young people with self-harmful thoughts.

Secondary Objectives: The secondary objectives are i) to investigate the impact of having access to Purrble on changes to LGBTQ+ young people's self-harmful thoughts over the trial period, in comparison to a waitlist control group; and ii) to investigate the impact of Purrble on changes in symptoms of anxiety (GAD-7), and symptoms of depression (PHQ-9) over the trial period, in comparison to waitlist controls. Finally, this will be the first opportunity to assess whether Purrble remains appealing and helpful to LGBTQ+ youth over an extended period.

Methods

Trial design

The study is a 2-arm randomised controlled trial comparing an intervention group (Purrble) with a waitlist control group. The trial period is across 13 weeks, built of 3 pre-deployment assessments and 10 deployment assessments, using weekly, self-reported, validated surveys (see table 1). The intervention period will commence once Purrble has been deployed to the intervention group, week 4 (T1). After the deployment period, the intervention participants will be able to keep their Purrble devices, and the waitlist control participants will be sent Purrble devices.

Table 1: Overview of assessment design for both participant groups

Surveys	Pre-deployment (week 1-3)	Deployment (week 4-13)	Follow-up (week 13+)

	T(-2)	T(-1)	T(0)	T1	T2	T3	T4	T5	T6	T7	T8	T9	T10	
Register interest + screening	X													
Consent	x													
Main assessment	x	x		x	x	x	x		x	x	x	x		
Extended assessment			x					x					x	
Qualitative interviews														x

For peer review only

Analyses will be conducted and reported in accordance with the Consolidated Standards of Reporting Trials (CONSORT; [51]), with consideration given to the recommendation of psychological interventions [52]. Outcomes will be assessed 13 times across a 13-week period, including 3 baseline assessments and 10 weeks of deployment, with Purrble being delivered in time for week 4 (T1).

Intervention

The intervention takes the form of an interactive plush toy (figure 1), which was co-designed with children to support in-the-moment soothing [48-49]. The device is framed as an anxious creature, in need of care and attention when it feels distressed. This distress is indicated by a simulated heartbeat using embedded electronics to produce vibration patterns of i) frantic and anxious, to ii) slow, steady and relaxed. When held, the device emits a frantic heartbeat which can be slowed by stroking movements registered by embedded sensors. Once the device has been "soothed" for long enough, the heartbeat transitions into a purring vibration, indicating a relaxed state. This transition can be achieved in less than 60 seconds but is

For peer review only - http://bmjopen.bmj.com/site/about/guidelines.xhtml

dependent on the device-human interaction. Further details on the logic model underlying Purrble can be found in supplementary materials 1, or see previous research [49].

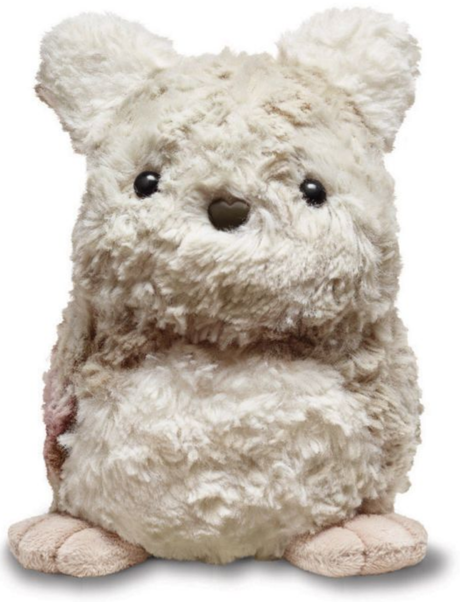

Figure 1: Purrble companion device

Waitlist - Control

The participants in the control group will be on a waitlist throughout the 13-week trial period. Once data have been collected at the final timepoint (week 13, T10), waitlist participants will receive a Purrble to keep. Waitlist control group was selected following discussions with Sprouting Minds members (see Patient and Public Involvement).

Participants

Eligibility Criteria

When potential participants register their interest for the study, they will be asked demographic questions relating to the eligibility criteria, providing our information for our inclusion criteria. These are: i) being between the ages of 16-25 years (inclusive); ii) identifying as any part of the LGBTQ+ umbrella, iii) having current experiences of self-harmful thoughts (e.g. in the last month), iv) being able to read, write, and speak English, and v) living in the UK for the duration of the study. Exclusion criteria are being outside of the

For peer review only - http://bmjopen.bmj.com/site/about/guidelines.xhtml

age range, identifying as heterosexual cisgender, not experiencing current self-harmful thoughts, being unable to understand English and living outside the UK.

Recruitment, Randomisation, and Blinding

LGBTQ+ youth will be recruited to the trial by several strategies. These include: i) approaching secondary schools and colleges, ii) social media adverts, iii) advertising through stakeholder charities and organisations (e.g., Bounce Black, Harmless, King's College London newsletter), and iv) online platforms (e.g., MQ Participate). Those organisations which involve gatekeepers (e.g., schools, colleges, charities, organisations) will be emailed by one of the leading researchers offering an introductory meeting to discuss the outline of study, explaining safeguarding protocols, and how to share this information with young people. Young LGBTQ+ people will then be able to register their interest in the study, anonymously from gatekeepers. At recruitment, participants will not be blinded to the fact that Purrble was designed to support ER. The information sheet specifies that participants will be asked about mood, self-harm, and ER over the course of the study.

Once eligibility is confirmed and young LGBTQ+ people have provided written informed consent, they will be 1:1 randomly assigned to either intervention or waitlist control group, using a computerised algorithm. A stratification procedure will be applied to balance gender identity (transgender and gender diverse youth vs. cisgender) across the two arms. The researcher conducting randomisation will be blind to treatment group allocation. However, the leading researcher and other team members who will be conducting safeguarding will be aware of group allocation. Researchers collecting outcome measures will be blinded to group allocation. Participants will be informed about their assigned condition. The intervention group will receive Purrble devices before T1 data are collected, with waitlist participants receiving their Purrble devices after the 3-month follow-up data collection.

In the event of change of circumstance, such as a serious adverse event, participants are asked to inform the research team. Specifically, for follow-up engagements a standard operation protocol which asks that if a situation changes for a young person (e.g., they are hospitalised) that the young person informs the research team. This will be directly asked ("Has your situation changed at all which might impact how you'd like to engage with the

For peer review only - http://bmjopen.bmj.com/site/about/guidelines.xhtml

study?") via email, with the understanding that the research team will have time to arrange reasonable adjustments (e.g., if a young person still wants to take part but is within an in-patient service).

Power Analysis

Sample size was determined based on an a-priori power analysis to detect a difference between the two arms when considering the primary outcome measure, the Difficulties with Emotion Regulation Scale-8 (DERS8). On the basis of a pilot with LGBTQ+ youth [47] and other Purrble studies [50, 53], we expect to see a medium effect size for this measure (d=0.4). This would indicate fewer difficulties with ER among young people who had access to Purrble.

With the anticipated medium effect size, simulations were performed involving a range of fixed and random effects. Simulations involved linearly increasing effect over the study period, and sensitivity analysis was performed over a range of scenarios considering the slope of effect change over time was either fixed or random. The simulation used a one sided t-test ($\propto$=0.05), and targeted a sample size giving at least 80% power. The statistic to be compared between groups is the change in mean DERS8 score in the 2 weeks preceding intervention (3 assessments in total), to the mean DERS8 assessed at weeks 8, 9, and 10. The averaging over 3 assessments is intended to reduce the known variance in DERS8 when repeatedly assessed [54]. Other simulations considered only comparing the change in DERS8 from baseline (week 0) to week 10, comparing the slope of the effect, assessed using simple linear regression. All simulations suggested better than 60% power with 70 subjects per arm, with the mean change in DERS8 averaged over 3 assessments, and the comparison of slopes, suggesting >80% power. The sample size of 140 is inflated by 20% to account for dropout rate [55], rounding up the total sample to 168.

Outcome Measures

An overview of all measures can be found in Table 2, and full details can be found in supplementary material 2. Primary and mental health measures will be asked at all timepoints, with the Purrble intervention group also receiving two additional engagement

measures throughout deployment. An extended survey will replace the weekly survey at three time points, this will include three additional measures to be asked to all participants.

These additional measures were selected to explore the association between self-harm and emotion regulation (Process Model Emotion Regulation Questionnaire - PMERQ) [38-41], perceptions of hope (State Hope Scale - SHS) [13], and loneliness (3-item UCLA loneliness scale) [56], to further explore the qualitative findings represented in our pilot study (Williams et al., in prep). Our findings have previously indicated that Purrble was used to i) refocus or distract attention during moments of distress by addressing the physical manifestation of their discomfort (PMERQ), and ii) comfort in moments of loneliness and provide self-soothing mechanisms (UCLA, SHS) [47].

Considering participant burden, young people will be informed of the time to complete each weekly survey (15 minutes), the extended survey (22 minutes) and will be compensated for their time.

Table 2: Summary of the outcome measures

Outcome Measure	Questions (n)	Type of outcome	Frequency	Scoring	Details of assessment
Primary measure					
Difficulties in emotion regulation scale-8 DERS8; [54]	8	Primary outcome	All timepoints	8-40 (higher = more difficulties)	Difficulties associated with response to situations eliciting negative emotions.

For peer review only

Mental health measures					
Self-Harm Questionnaire screening questions<br><br>SHQ; [57]	3	Mental health	All timepoints	Analysed separately	Frequency and risk of self-harm thoughts, suicidal ideation, and behaviour.
Self-Harm Questionnaire additional items<br><br>SHQ; [57]	22	Covariate	Once (baseline)	Analysed separately	4 dimensions of self-harm (NSSI, suicide attempts, suicide threats, suicide ideation).
Generalised Anxiety Disorder questionnaire<br><br>GAD-7; [58]	7	Mental health	All timepoints	0-21 (higher = greater severity)	Presence and severity of generalized anxiety disorder
Patient Health Questionnaire<br><br>PHQ-9; [59]	9	Mental health	All timepoints	0-27 (higher = greater severity)	Severity of depressive symptoms
Proximal & mechanistic measures					

For peer review only - http://bmjopen.bmj.com/site/about/guidelines.xhtml

Process Model of Emotion Regulation Questionnaire<br><br>PMERQ; [60]	9	Mechanistic	3 timepoints	2 subscales<br><br>Average across each subscale, with higher scores indicating greater endorsement.	Attentional deployment subscales are selected; engagement and disengagement focus.
State Hope Scale<br><br>SHS; [61]	6	Proximal	3 timepoints	6-48 (higher = greater state hopefulness)	Ongoing goal-directed thinking; agency and pathways.
The 3-item UCLA Loneliness scale for children<br><br>[62]	3	Proximal	3 timepoints	3-12 (higher = more loneliness)	Subjective feelings of loneliness.
Engagement measures					
Bespoke Purrble questions<br><br>[53]	7	Engagement	Deployment	Analysed separately	Purrble use and perceived usefulness.

For peer review only

Twente Engagement With eHealth Technologies Scale TWEETS; [63]	9	Engagement	Deployment	3 subscales Total score per subscale = greater engagement	Engagement with intervention device, considering behaviour, cognitive, and affective engagements.

Post deployment Interviews

We will collect semi-structured interview data from LGBTQ+ young people from up to 40% of the intervention group (n=37), and approximately 20% of the control group (n=17). The interviews will be conducted following the deployment period. We will specifically aim to recruit young people who demonstrated the highest and lowest changes in the outcome data over the trial to explore and understand the potential moderators relating to the intervention and mental health across the trial period.

The semi-structured interview will explore the engagement and appropriation of the Purrble device, whether LGBTQ+ young people had felt that this had helped them with their ER, mental health more broadly, or self-harmful thoughts, and how Purrble may (or may not) be suitable for other audiences. We will compare these experiences between intervention and control participants, exploring other mechanisms used by LGBTQ+ youth who experience self-harm.

Hypotheses

Primary hypothesis

Across the trial, we hypothesise that access to the Purrble intervention (compared to the waitlist control) will lead to a direct decrease in self-reported difficulties with emotion

For peer review only - http://bmjopen.bmj.com/site/about/guidelines.xhtml

regulation as measured by the primary outcome (DERS8), averaged between three pre-deployment (weeks 1-3) and our final three deployment assessments (weeks 11-13).

Secondary hypothesis

Intervention effects will be moderated by engagement with the device, measured by bespoke questions and the TWEETS questionnaire. Secondary outcomes in the Purrble effectiveness trial are: self-harmful thoughts, symptoms of anxiety, and symptoms of depression. These three constructs were selected as secondary outcomes based on the high prevalence of these experiences among LGBTQ+ youth [3-8] and their association with poor ER [38-41]. The three secondary hypotheses are as listed when compared to waitlist controls:

1. Engagement with the Purrble intervention will reduce the frequency of self-reported self-harmful thoughts (SHQ).
2. Engagement with the Purrble intervention will reduce the severity of self-reported anxiety symptoms (GAD-7).
3. Engagement with the Purrble intervention will reduce the severity of self-reported depression symptoms (PHQ-9).

Additional analyses

Additional hypotheses aim to understand the impact of Purrble on relevant proximal and mechanistic outcomes. The following hypotheses will be investigated across the trial:

1. Greater within-group changes will be seen among intervention group participants, with increasing levels of endorsement for attentional deployment (PMERQ), than among those participants of the control group.
2. There will be a greater increase in state hopefulness (SHS) in the Purrble intervention group than the waitlist control.
3. Participants in the Purrble intervention group will report lower loneliness (UCLA) than those in the waitlist control group.

For peer review only - http://bmjopen.bmj.com/site/about/guidelines.xhtml

Statistical analyses

Testing the hypothesis that access to the Purrble intervention will lead to a reduction in emotion dysregulation, as measured by the composite primary outcome, will be done using a one-sided t-test.

As exploratory analyses linear mixed models will be fitted to gain insight into how emotion dysregulation is altered with access to the intervention. In particular, we will regress the weekly outcome score on an indicator for the Purrble condition, a linear time trend, and an interaction between the treatment indicator and time to examine differential trends in the two groups. We will adjust for baseline covariates and include participant-level random intercepts and slopes to account for persistent baseline differences between young people as well as person-specific time trends in the outcome. While the outcome is limited to DERS8 scores ranging from 8-40, we will model it as continuous data.

For secondary aims, we will use analogous linear mixed models to assess the impact of Purrble on relevant outcomes (cf., hypotheses above), adding baseline DERS8 as another covariate. We will not adjust for multiple comparisons, as these are exploratory aims meant to be hypothesis-generating. Similarly, we will also assess the impact of access to Purrble on changes in proximal outcomes, as well as explore whether these appear to moderate changes on primary and secondary outcomes.

Patient and Public Involvement

The study design was discussed with, and approved by, Sprouting Minds members (MRC Digital Youth Young Person Advisory group), with specific input considering the intervention arms and safeguarding procedures. These young people highlighted that “waitlist control” conditions mimic clinical experiences of waiting for services, therefore this was considered an acceptable and realistic control. Whereas safeguarding procedures needed to balance participant autonomy and ensuring safe research.

For peer review only

Ethics and dissemination

The study will be conducted according to local regulations and the Declaration of Helsinki of 1975, revised in 2008. The Ethical Committee at King's College London, UK approved the study (RESCM-22/23-34570). Written consent will be obtained from all participants prior to commencing their involvement in the study, with explicit understanding of the study and safeguarding procedures (see supplementary materials 3) being obtained during study briefing sessions. The trial is registered with ClinicalTrivals.gov (NCT06025942).

We aim for our findings to be disseminated across academic fields (human-computer interactions, psychology, implementation sciences), alongside showcasing the findings to LGBTQ+ youth, community groups, and wider stakeholders. This will be achieved through presentations at national and international conferences, peer-reviewed journal publications, community outreach and PPI events. During dissemination, we will be liaising with youth populations to establish next steps for this research, considering additional co-design of materials to sit around/alongside Purrble.

Discussion

Despite the high prevalence of self-harm among LGBTQ+ young people [10, 37], there is little/no evidence for interventions which support the reduction of self-harm through situated skill application of ER. This low-cost intervention has the potential to reduce difficulties associated with emotion regulation, and therefore offer an opportunity to improve youth's self-harm, as well as other associated mental health difficulties. If successful, the study will provide a proof-of-concept example of a bottom-up emotion regulation intervention, enabling young people to access ongoing support for their distress through an engaging digital resource.

Previous research with severely anxious students has indicated that Purrble was associated with reductions of anxious symptoms, qualitatively explained by the adoption of ER strategies [50]. These findings were translated in our pilot study of Purrble within the LGBTQ+ population [47]. Therefore, it is hypothesised that in this study, engagement with Purrble will be associated with reduced anxiety and increased ER across the trial.

For peer review only - http://bmjopen.bmj.com/site/about/guidelines.xhtml

Effective ER, such as utilising adaptive strategies (e.g., cognitive appraisal), can be seen as a protective factor in self-harm and suicide research [64]. If Purrble can support reduction of self-harmful thoughts in young LGBTQ+ people through enhanced ER, then this is potentially a tangible, highly modular and scalable device which can be used to prevent the transition between motivational and volitional experiences [65].

Acknowledgements

The authors (AJW, ET, NN, ACN, CH, PS) acknowledge the support of the UK Research and Innovation (UKRI) Digital Youth Programme award [MRC project reference MR/W002450/1] which is part of the AHRC/ESRC/MRC Adolescence, Mental Health and the Developing Mind programme. The authors would like to thank our PPI group Sprouting Minds, including the two co-chairs, Sarah Doherty and Lucy-Paige Willingham, for their ongoing support and contributions to the project. RB receives salary and research support from an Australian National Health and Medical Research Council (NHMRC) Emerging Leadership Investigator Grant (EL2; GNT2008073).

AJW, ET, PS conceived the study. AJW and PS designed the study, with statistical expertise from CT and patient and public input from NN and ACN. AJW designed the study materials, which were reviewed with feedback between NN, ACN, and SC. AJW obtained ethical approval. JJG, ET, PS and AJW contributed to measure selection. AJW drafted the first version of the protocol manuscript for publication, with input from all authors. RB and AJW revised the submitted protocol manuscript together.

We would also like to thank Mathijs Lucassen for his input while developing this trial.

Funding

This study is funded by the UKRI MRC Digital Youth (MR/W002450/1) and the UKRI Future Leaders Fellowship (MR/T041897/1).

For peer review only

Competing interests statement

PS has been involved in the development of what is now Purrble as part of his postdoctoral fellowship; and serves as a paid research adviser to the Committee for Children but has no financial stake in either CfC (Purrble brand owners) or Sproutel (company manufacturing Purrbles). Neither Committee for Children nor Sproutel had access to the data or had been part of the data collection in any way, nor did they approve or see the publication before it was submitted. There were no conflicting interests among the remaining research team.

References:

1. Kendall T, Taylor C, Bhatti H, Chan M, Kapur N. Longer term management of self harm: summary of NICE guidance. Bmj. 2011 Nov 23;343. doi.org/10.1136/bmj.d7073
2. World Health Organisation. Suicide [fact sheet] [Internet]. World Health Organization; 2021 [cited 2023 Mar 24]. Available from: https://www.who.int/news-room/fact-sheets/detail/suicide
3. Coker TR, Austin SB, Schuster MA. The health and health care of lesbian, gay, and bisexual adolescents. Annual review of public health. 2010 Apr 21;31:457-77. doi.org/10.1146/annurev.publhealth.012809.103636
4. Haas AP, Eliason M, Mays VM, Mathy RM, Cochran SD, D'Augelli AR, Silverman MM, Fisher PW, Hughes T, Rosario M, Russell ST. Suicide and suicide risk in lesbian, gay, bisexual, and transgender populations: Review and recommendations. Journal of homosexuality. 2010 Dec 30;58(1):10-51. DOI: 10.1080/00918369.2011.534038
5. Marshal MP, Dietz LJ, Friedman MS, Stall R, Smith HA, McGinley J, Thoma BC, Murray PJ, D'Augelli AR, Brent DA. Suicidality and depression disparities between sexual minority and heterosexual youth: A meta-analytic review. Journal of adolescent health. 2011 Aug 1;49(2):115-23. doi.org/10.1016/j.jadohealth.2011.02.005
6. Saewyc EM. Research on adolescent sexual orientation: Development, health disparities, stigma, and resilience. Journal of research on adolescence. 2011 Mar;21(1):256-72. doi.org/10.1111/j.1532-7795.2010.00727.x
7. McDermott E, Hughes E, Rawlings V. Norms and normalisation: understanding lesbian, gay, bisexual, transgender and queer youth, suicidality and help-seeking.

For peer review only

Culture, Health & Sexuality. 2017 Feb 1;20(2):156-72. doi.org/10.1080/13691058.2017.1335435

8. Irish M, Solmi F, Mars B, King M, Lewis G, Pearson RM, Pitman A, Rowe S, Srinivasan R, Lewis G. Depression and self-harm from adolescence to young adulthood in sexual minorities compared with heterosexuals in the UK: a population-based cohort study. The Lancet Child & Adolescent Health. 2019 Feb 1;3(2):91-8. doi.org/10.1016/S2352-4642(18)30343-2
9. Chan MK, Bhatti H, Meader N, Stockton S, Evans J, O'Connor RC, Kapur N, Kendall T. Predicting suicide following self-harm: systematic review of risk factors and risk scales. The British Journal of Psychiatry. 2016 Oct;209(4):277-83. doi: 10.1192/bjp.bp.115.170050
10. Di Giacomo E, Krausz M, Colmegna F, Aspesi F, Clerici M. Estimating the risk of attempted suicide among sexual minority youths: a systematic review and meta-analysis. JAMA pediatrics. 2018 Dec 1;172(12):1145-52. doi:10.1001/jamapediatrics.2018.2731
11. Marchi M, Arcolin E, Fiore G, Travascio A, Uberti D, Amaddeo F, Converti M, Fiorillo A, Mirandola M, Pinna F, Ventriglio A. Self-harm and suicidality among LGBTIQ people: a systematic review and meta-analysis. International review of psychiatry. 2022 May 19;34(3-4):240-56. doi.org/10.1080/09540261.2022.2053070
12. Warne N, Heron J, Mars B, Solmi F, Biddle L, Gunnell D, Hammerton G, Moran P, Munafò M, Penton-Voak I, Skinner A. Emotional dysregulation in childhood and disordered eating and self-harm in adolescence: prospective associations and mediating pathways. Journal of child psychology and psychiatry. 2023 May;64(5):797-806. doi.org/10.1111/jcpp.13738
13. Mars B, Heron J, Klonsky ED, Moran P, O'Connor RC, Tilling K, Wilkinson P, Gunnell D. Predictors of future suicide attempt among adolescents with suicidal thoughts or non-suicidal self-harm: a population-based birth cohort study. The Lancet Psychiatry. 2019 Apr 1;6(4):327-37. doi.org/10.1016/S2215-0366(19)30030-6
14. Geulayov G, Casey D, McDonald KC, Foster P, Pritchard K, Wells C, Clements C, Kapur N, Ness J, Waters K, Hawton K. Incidence of suicide, hospital-presenting non-fatal self-harm, and community-occurring non-fatal self-harm in adolescents in England (the iceberg model of self-harm): a retrospective study. The Lancet Psychiatry. 2018 Feb 1;5(2):167-74. doi.org/10.1016/S2215-0366(17)30478-9

For peer review only - http://bmjopen.bmj.com/site/about/guidelines.xhtml

15. Geulayov G, Borschmann R, Mansfield KL, Hawton K, Moran P, Fazel M. Utilization and acceptability of formal and informal support for adolescents following self-harm before and during the first COVID-19 lockdown: results from a large-scale English schools survey. Frontiers in psychiatry. 2022 Jun 24;13:881248. doi.org/10.3389/fpsyt.2022.881248
16. Holland J, Sayal K, Berry A, Sawyer C, Majumder P, Vostanis P, Armstrong M, Harroe C, Clarke D, Townsend E. What do young people who self-harm find helpful? A comparative study of young people with and without experience of being looked after in care. Child and adolescent mental health. 2020 Sep;25(3):157-64. Doi.org/10.1111/camh.12384
17. Michelmore L, Hindley P. Help-seeking for suicidal thoughts and self-harm in young people: A systematic review. Suicide and Life-Threatening Behavior. 2012 Oct;42(5):507-24. doi.org/10.1111/j.1943-278X.2012.00108.x
18. Owens C, Hansford L, Sharkey S, Ford T. Needs and fears of young people presenting at accident and emergency department following an act of self-harm: secondary analysis of qualitative data. The British Journal of Psychiatry. 2016 Mar;208(3):286-91. doi.org/10.1192/bjp.bp.113.141242
19. Williams AJ, Nielsen E, Coulson NS. “They aren’t all like that”: Perceptions of clinical services, as told by self-harm online communities. Journal of health psychology. 2020 Nov;25(13-14):2164-77. doi.org/10.1177/13591053187884
20. Long M, Manktelow R, Tracey A. We are all in this together: working towards a holistic understanding of self-harm. Journal of Psychiatric and Mental Health Nursing. 2013 Mar;20(2):105-13. doi.org/10.1111/j.1365-2850.2012.01893.x
21. Bathje G, Pryor J. The relationships of public and self-stigma to seeking mental health services. Journal of Mental Health Counseling. 2011 Apr 1;33(2):161-76. doi.org/10.17744/mehc.33.2.g6320392741604l1
22. Stonewall. LGBT in Britain Health Report [Internet]. Stonewall; 2018 [cited 2023 Mar 24]. Available from: https://www.stonewall.org.uk/system/files/lgbt_in_britain_health.pdf
23. Steinke J, Root-Bowman M, Estabrook S, Levine DS, Kantor LM. Meeting the needs of sexual and gender minority youth: formative research on potential digital health interventions. Journal of Adolescent Health. 2017 May 1;60(5):541-8. doi.org/10.1016/j.jadohealth.2016.11.023

For peer review only - http://bmjopen.bmj.com/site/about/guidelines.xhtml

24. Bowen D, Jabson J, Kamen C. mHealth: an avenue for promoting health among sexual and gender minority populations?. Mhealth. 2016;2. doi: 10.21037/mhealth.2016.09.01
25. Strauss P, Morgan H, Toussaint DW, Lin A, Winter S, Perry Y. Trans and gender diverse young people's attitudes towards game-based digital mental health interventions: A qualitative investigation. Internet Interventions. 2019 Dec 1;18:100280. Doi.org/10.1016/j.invent.2019.100280
26. Craig SL, McInroy L. You can form a part of yourself online: The influence of new media on identity development and coming out for LGBTQ youth. Journal of Gay & Lesbian Mental Health. 2014 Jan 1;18(1):95-109. doi.org/10.1080/19359705.2013.777007
27. Gulliver A, Griffiths KM, Christensen H. Perceived barriers and facilitators to mental health help-seeking in young people: a systematic review. BMC psychiatry. 2010 Dec;10(1):1-9. doi.org/10.1186/1471-244X-10-113
28. McIntyre J, Daley A, Rutherford K, Ross LE. Systems-level barriers in accessing supportive mental health services for sexual and gender minorities: Insights from the provider's perspective. Canadian Journal of Community Mental Health. 2012 Mar 22;30(2):173-86. doi.org/10.7870/cjcmh-2011-0023
29. Gilbey D, Morgan H, Lin A, Perry Y. Effectiveness, acceptability, and feasibility of digital health interventions for LGBTIQ+ young people: systematic review. Journal of medical Internet research. 2020 Dec 3;22(12):e20158. doi:10.2196/20158
30. Martin S. *Developing and evaluating QueerViBE: an online intervention to empower trans and non-binary youth* (Doctoral dissertation, Anglia Ruskin University). [cited 2023 Mar 24].
31. Pachankis JE, Goldfried MR. Expressive writing for gay-related stress: psychosocial benefits and mechanisms underlying improvement. Journal of consulting and clinical psychology. 2010 Feb;78(1):98. Doi.org/10.1037/a0017580
32. Lucassen MF, Merry SN, Hatcher S, Frampton CM. Rainbow SPARX: A novel approach to addressing depression in sexual minority youth. Cognitive and Behavioral Practice. 2015 May 1;22(2):203-16. Oi.org/10.1016/j.cbpra.2013.12.008
33. Fleming JB, Hill YN, Burns MN. Usability of a culturally informed mHealth intervention for symptoms of anxiety and depression: feedback from young sexual minority men. JMIR human factors. 2017 Aug 25;4(3):e7392. doi: 10.2196/humanfactors.7392

For peer review only - http://bmjopen.bmj.com/site/about/guidelines.xhtml

34. Fish JN, Williams ND, McInroy LB, Paceley MS, Edsall RN, Devadas J, Henderson SB, Levine DS. Q Chat Space: assessing the feasibility and acceptability of an internet-based support program for LGBTQ youth. Prevention science. 2021 Sep 6:1-2. doi.org/10.1007/s11121-021-01291-y

35. Williams AJ, Arcelus J, Townsend E, Michail M. Feasibility and acceptability of experience sampling among LGBTQ+ young people with self-harmful thoughts and behaviours. Frontiers in psychiatry. 2022 Aug 17;13:916164. Doi.org/10.3389/fpsyt.2022.916164

36. Williams AJ, Arcelus J, Townsend E, Michail M. Understanding the processes underlying self-harm ideation and behaviors within LGBTQ+ young people: A qualitative study. Archives of suicide research. 2021 Apr 3;27(2):380-96. doi.org/10.1080/13811118.2021.2003273

37. Hatchel T, Polanin JR, Espelage DL. Suicidal thoughts and behaviors among LGBTQ youth: Meta-analyses and a systematic review. Archives of suicide research. 2019 Jan 2;25(1):1-37. doi.org/10.1080/13811118.2019.1663329

38. Chen WL, Chun CC. Association between emotion dysregulation and distinct groups of non-suicidal self-injury in Taiwanese female adolescents. International journal of environmental research and public health. 2019 Sep;16(18):3361. Doi.org/10.3390/ijerph16183361

39. Clapham R, Brausch A. Internalizing and externalizing symptoms moderate the relationship between emotion dysregulation and suicide ideation in adolescents. Child Psychiatry & Human Development. 2022 Sep 6:1-2. doi.org/10.1007/s10578-022-01413-9

40. Kapatais A, Williams AJ, Townsend E. The mediating role of emotion regulation on self-harm among gender identity and sexual orientation minority (LGBTQ+) individuals. Archives of suicide research. 2023 Apr 3;27(2):165-78. doi.org/10.1080/13811118.2022.2064254

41. Fraser G, Wilson MS, Garisch JA, Robinson K, Brocklesby M, Kingi T, O'Connell A, Russell L. Non-suicidal self-injury, sexuality concerns, and emotion regulation among sexually diverse adolescents: A multiple mediation analysis. Archives of suicide research. 2018 Jul 3;22(3):432-52. doi.org/10.1080/13811118.2017.1358224

42. Sloan E, Hall K, Moulding R, Bryce S, Mildred H, Staiger PK. Emotion regulation as a transdiagnostic treatment construct across anxiety, depression, substance, eating

For peer review only

and borderline personality disorders: A systematic review. Clinical psychology review. 2017 Nov 1;57:141-63. Doi.org/10.1016/j.cpr.2017.09.002

43. Gross JJ. Emotion regulation: Current status and future prospects. Psychological inquiry. 2015 Jan 2;26(1):1-26. doi.org/10.1080/1047840X.2014.940781
44. Fernandez KC, Jazaieri H, Gross JJ. Emotion regulation: A transdiagnostic perspective on a new RDoC domain. Cognitive therapy and research. 2016 Jun;40:426-40. doi.org/10.1007/s10608-016-9772-2
45. Wolff JC, Thompson E, Thomas SA, Nesi J, Bettis AH, Ransford B, Scopelliti K, Frazier EA, Liu RT. Emotion dysregulation and non-suicidal self-injury: A systematic review and meta-analysis. European Psychiatry. 2019 Jun;59:25-36. Doi.org/10.1016/j.eurpsy.2019.03.004
46. Fox KR, Franklin JC, Ribeiro JD, Kleiman EM, Bentley KH, Nock MK. Meta-analysis of risk factors for nonsuicidal self-injury. Clinical psychology review. 2015 Dec 1;42:156-67. Doi.org/10.1016/j.cpr.2015.09.002
47. Williams, AJ, Townsend, T, Naeche, N, Chapman-Nisar, A, Hollis, C, Slovak, P. Investigating the feasibility, acceptability and appropriation of a socially assistive robot among minority youth at-risk of self-harm. [in submission]
48. Slovák P, Theofanopoulou N, Cecchet A, Cottrell P, Altarriba Bertran F, Dagan E, Childs J, Isbister K. " I just let him cry... Designing Socio-Technical Interventions in Families to Prevent Mental Health Disorders. Proceedings of the ACM on Human-Computer Interaction. 2018 Nov 1;2(CSCW):1-34. Doi.org/10.1145/3274429
49. Theofanopoulou N, Isbister K, Edbrooke-Childs J, Slovák P. A smart toy intervention to promote emotion regulation in middle childhood: Feasibility study. JMIR mental health. 2019 Aug 5;6(8):e14029. doi: 10.2196/14029
50. Williams AJ, Freed M, Theofanopoulou N, Roquet CD, Klasnja P, Gross JJ, Schleider J, Slovak P. Feasibility, perceived impact, and acceptability of a socially assistive robot to support emotion regulation with highly anxious university students: an open trial. JMIR Mental Health. 2023. [in prep]
51. Schulz KF, Altman DG, Moher D, CONSORT Group*. CONSORT 2010 statement: updated guidelines for reporting parallel group randomized trials. Annals of internal medicine. 2010 Jun 1;152(11):726-32. doi.org/10.7326/0003-4819-152-11-201006010-00232

For peer review only

For peer review only - http://bmjopen.bmj.com/site/about/guidelines.xhtml

52. Montgomery P, Grant S, Mayo-Wilson E, Macdonald G, Michie S, Hopewell S, Moher D. Reporting randomised trials of social and psychological interventions: the CONSORT-SPI 2018 Extension. Trials. 2018 Dec;19(1):1-4. doi.org/10.1186/s13063-018-2733-1
53. Daudén Roquet C, Theofanopoulou N, Freeman JL, Schleider J, Gross JJ, Davis K, Townsend E, Slovak P. Exploring Situated & Embodied Support for Youth's Mental Health: Design Opportunities for Interactive Tangible Device. In Proceedings of the 2022 CHI Conference on Human Factors in Computing Systems 2022 Apr 29 (pp. 1-16). doi.org/10.1145/3491102.3502135
54. Penner F, Steinberg L, Sharp C. The Development and Validation of the Difficulties in Emotion Regulation Scale-8: Providing Respondents with a Uniform Context That Elicits Thinking About Situations Requiring Emotion Regulation. Journal of Personality Assessment. 2022 Oct 7:1-0. Doi.org/10.1080/00223891.2022.2133722
55. Cohen KA, Schleider JL. Adolescent dropout from brief digital mental health interventions within and beyond randomized trials. Internet Interventions. 2022 Mar 1;27:100496. Doi.org/10.1016/j.invent.2022.100496
56. Shaw RJ, Cullen B, Graham N, Lyall DM, Mackay D, Okolie C, Pearsall R, Ward J, John A, Smith DJ. Living alone, loneliness and lack of emotional support as predictors of suicide and self-harm: A nine-year follow up of the UK Biobank cohort. Journal of Affective Disorders. 2021 Jan 15;279:316-23. Doi.org/10.1016/j.jad.2020.10.026
57. Ougrin D, Boege I. Brief report: the Self Harm Questionnaire: a new tool designed to improve identification of self harm in adolescents. Journal of adolescence. 2013 Feb 1;36(1):221-5. Doi.org/10.1016/j.adolescence.2012.09.006
58. Spitzer RL, Kroenke K, Williams JB, Löwe B. A brief measure for assessing generalized anxiety disorder: the GAD-7. Archives of internal medicine. 2006 May 22;166(10):1092-7. doi:10.1001/archinte.166.10.1092
59. Kroenke K, Spitzer RL, Williams JB. The PHQ-9: validity of a brief depression severity measure. Journal of general internal medicine. 2001 Sep;16(9):606-13. doi.org/10.1046/j.1525-1497.2001.016009606.x
60. Olderbak S, Uusberg A, MacCann C, Pollak KM, Gross JJ. The Process Model of Emotion Regulation Questionnaire: assessing individual differences in strategy stage and orientation. Assessment. 2022 Dec 9:10731911221134601.

For peer review only - http://bmjopen.bmj.com/site/about/guidelines.xhtml

61. Snyder CR, Sympson SC, Ybasco FC, Borders TF, Babyak MA, Higgins RL. Development and validation of the State Hope Scale. Journal of personality and social psychology. 1996 Feb;70(2):321. doi.org/10.1037/0022-3514.70.2.321
62. Testing of loneliness questions in surveys - Office for National Statistics [Internet]. Ons.gov.uk. 2018. Available from: https://www.ons.gov.uk/peoplepopulationandcommunity/wellbeing/compendium/nationalmeasurementofloneliness/2018/testingoflonelinessquestionsinsurveys. [cited 2023 Mar 24].
63. Kelders SM, Kip H, Greeff J. Psychometric evaluation of the TWente Engagement with Ehealth Technologies Scale (TWEETS): evaluation study. Journal of medical internet research. 2020 Oct 9;22(10):e17757. doi: 10.2196/17757
64. Colmenero-Navarrete L, García-Sancho E, Salguero JM. Relationship between emotion regulation and suicide ideation and attempt in adults and adolescents: a systematic review. Archives of suicide research. 2022 Oct 2;26(4):1702-35. Doi.org/10.1080/13811118.2021.1999872
65. O'Connor RC, Kirtley OJ. The integrated motivational–volitional model of suicidal behaviour. Philosophical Transactions of the Royal Society B: Biological Sciences. 2018 Sep 5;373(1754):20170268. doi.org/10.1098/rstb.2017.0268

For peer review only

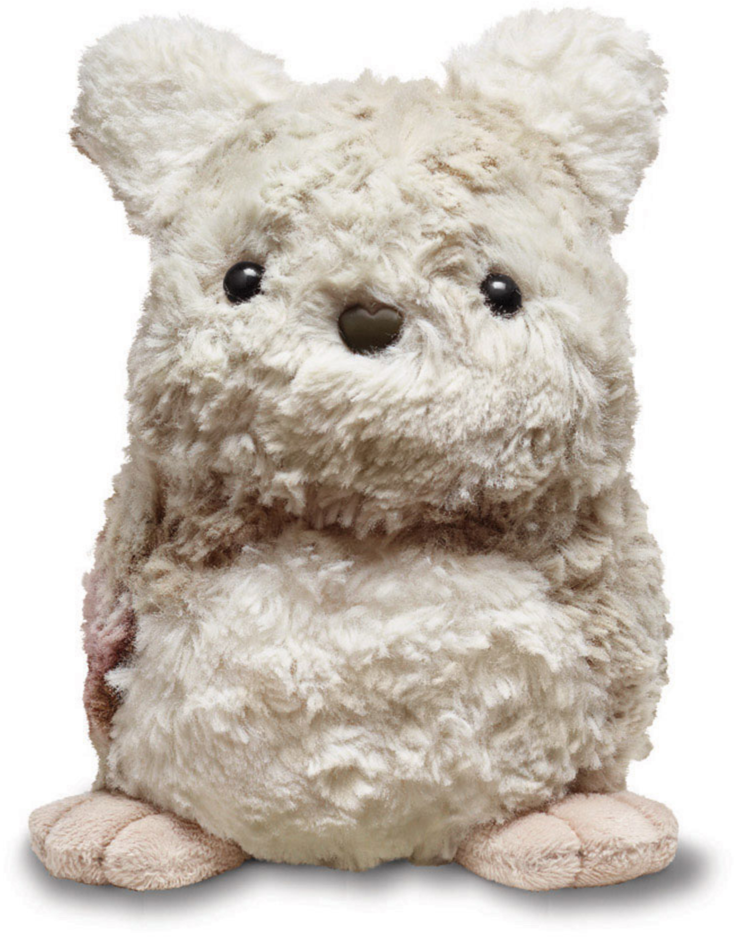

Purrble: Intervention device

352x349mm (72 x 72 DPI)

Purrble logic model

Purrble operates on 3 levels:

1. Purrble directly provides in-the-moment soothing support in naturally occurring emotional moments when one would attempt to calm down. The physical and interactive design aims to tap into known regulatory factors using the extended Process Model of Emotion Regulation (Gross, 2015). This focuses on two separate stages of the ER process; i) the attentional deployment stage (Aldao et al., 2015; Sheppes & Gross, 2012; Sheppes et al., 2014; Farb et al., 2014) by shifting attention from the emotional situation towards interacting with the device, and ii) the response modulation stage (Beetz et al., 2012; Dore et al., 2017; Reeck et al., 2016; Coan et al., 2006; Crossman et al., 2018; Rabbit et al., 2015) by facilitating down-regulation through pleasant tactical interactions simulating the emotion regulatory effect of human-animal interactions.

2. Mechanisms of Purrble are designed to facilitate long-term engagement, by building on positive subjective experiences of in-the-moment soothing. As the device is framed as an anxious creature needing to be cared for, the key driver is that interactions are framed as helping regulate others' emotions (Dore et al., 2017; Cosley et al., 2010; Taylor, 2011), alongside facilitating a sense of relationship and responsibility for the well-being of the creature (Turkle, 2007; Hayashi & Kato, 2016; Donath, 2004; Lee et al., 2010).

3. Through repeated, soothing, and positive interactions with Purrble over time, it is anticipated that there will be a shift in ER practices and implicit beliefs about emotions (Ford & Gross, 2018), specifically the controllability of one's emotions (Schleider & Weisz, 2016).

For peer review only

Full Measurement Details

Primary Outcome Measure

The primary outcome measure in this study is the Difficulties with Emotion Regulation Scale-8 (DERS8) [54]. This will be given to participants as part of the main assessment at all time-points. The DERS8 contains 8 items which represent affect, thoughts, and actions in response to situations which elicit negative emotions. A single total score is calculated from all items, with higher scores indicating more difficulties with emotion.

Mental Health Measures

Self-harmful thoughts and behaviours will be assessed using the Self-Harm Questionnaire (SHQ) [57]. At baseline, those who indicate experiences of self-harm behaviour will be invited to complete the second section about historical self-harm behaviour. At all other timepoints, only the three screening questions will be presented; these query the frequency of self-harmful thoughts, suicidal ideation, and self-harmful behaviour. The items are scored individually from "no thoughts/behaviour" to "yes, five or more times". The wording for these items will be adapted from "have you ever…" to "in the last week/month" (depending on assessment point).

Anxiety symptoms will be assessed using the Generalised Anxiety Disorder-7 (GAD-7) [58]. This is a seven-item instrument, used to identify or assess the severity of generalised anxiety disorder. Each item asks the individual to rate the severity of their symptoms over the time period. The total score is calculated by summing all items and ranges from 0-21. Higher scores indicate more severe levels of anxiety symptoms. The wording for these items will be adapted from "over the last two weeks" to "in the last week/month" (depending on assessment point).

Depressive symptoms will be assessed using the Patient Health Questionnaire-9 (PHQ9) [59]. Across nine items, this instrument measures the severity of depressive symptoms, the total score being calculated by summing all items with responses ranging from 0-27. Higher scores indicate greater depressive severity. Again, the wording for these items will be adapted from "over the last two weeks" to "in the last week/month" (depending on assessment point).

Proximal & Mechanistic Measures

All proximal and mechanistic measures will only be asked at three timepoints (T0, T5, T10), this is to reduce participant burden and encourage engagement, while also obtaining exploratory data to understand the impact of access to Purrble in greater detail. All additional measures total to 18 extra items, adding ~7 minutes to the assessment.

The Process Model of Emotion Regulation Questionnaire [60] is a 45-item measure which considers 10 ER strategies across the five stages of the Process Model of ER, and particularly how these strategies are used to decrease negative emotions. We will include 2 subscales focusing on attention deployment (focusing elsewhere (4 items) and cognitive distraction (5 items)). Each subscale is scored by taking the average of item-level responses.

For peer review only

Hopefulness will be assessed using the State Hope Scale [61]. This is a six-item instrument concerning ongoing goal-directed thinking (agency and pathways). The total score (6-48) is calculated by summing responses, with higher scores relating to greater state hopefulness.

Loneliness is assessed using the 3-item UCLA loneliness scale for children [62] to consider participants' subjective loneliness. Items are summed to create a total score (3-12), whereby higher results indicate greater loneliness.

Engagement Measures

Engagement with Purrble will be assessed using two measures; a bespoke survey [53] and an adapted version of Twente Engagement with eHealth Technologies Scale (TWEETS) [63]. These will be deployed as part of the main assessment for participants allocated to the intervention group.

The bespoke survey [53] enquires about Purrble use and perceived usefulness over four items. These are analysed separately, with qualitative responses indicating contexts or situations where Purrble has been found helpful or unhelpful.

TWEETS [63] quantitatively measures intervention engagement across nine items. This is split into subsections considering behaviour, cognitive, and affective engagement. Total scores range from 0-36, with higher scores indicating greater engagement.

For peer review only

Safeguarding Procedures

The safeguarding procedures will be presented to potential participants in the information sheet before they consent to the study. These were codesigned and agreed with Sprouting Minds, based on previous studies conducted with young people with self-harmful experiences (Williams et al., 2021; Williams et al., 2022; Williams et al., in prep; Townsend et al., 2016). To support young people's safety during the study, the following measures will be instituted:

1. Before starting the study, all participants are required to attend an individual study briefing. At this point, they are taken through the study outline, invited to ask questions, and asked to create a safety plan (Brown & Stanley, 2012) with the support of a specially trained researcher. Safety planning is quick to complete, is tangible to participants and has been shown to be effective at reducing self-harmful thoughts and behaviours (Nuiji et al., 2021). Participants keep this safety plan containing their individualised strategies for their personal use beyond the life of the study. During the wellbeing check, participants are asked to reflect on their safety plan if they feel distressed at any other point during the study. This follows safety protocols used in other studies by the researcher (Williams et al., 2021; Williams et al., 2022, pilot studies).
2. During the briefing, participants will need to nominate a support contact (parent, friend over 18 years, GP, etc), with the understanding that if they do not respond to reactive safeguarding (which can be triggered for each of the main assessments), this person will be contacted to ensure that the participant is safe.
    a. This nominated person will be contacted (via email) to inform them that the participant is taking part in a mental health study and that we will reach out if we are unable to contact the participant following indication that they are at increased risk.
3. All assessments will include signposting to additional supports (e.g., Young Minds, Samaritans, Papyrus) as well as encouraging participants to seek help from their GP if they are distressed.
4. All assessments will include a visual scale to rate mood pre- and post- completing the survey (1 - very distressed - 10 extremely happy). This is to examine whether the assessment process has an impact, positively or negatively, on the participant. Such assessments have been successfully used in prior self-harm research by the research team members as an indicator of assessment impact (Townsend et al., 2016; Williams et al., 2021).
5. Participants in need of support will be identified by checking their responses to the main battery of assessments, within 24 hours. If participants respond that they have had experiences of self-harmful or suicidal thoughts and self-harm behaviour, as well as showing a that the assessment has had a negative impact, they receive a wellbeing call the following day between 1pm-4pm. This allows enough time for the researcher to identify participant risk and inform the PI.
    a. If the participant does not pick up the phone, they will be contacted via email, asking about their wellbeing, whether they wish to continue with the study, and to arrange a time convenient to complete a wellbeing check. During the wellbeing check, the researcher will be empathic to the difficulties of the participant. The participant will be asked whether they wish to continue the study and, if so, they will update their safety plan with the researcher. If participants do not wish to continue the study, they will be allowed to withdraw with no consequences. They will be invited to interview to discuss their

For peer review only

thoughts, opinions and experiences of the study. This would also ask about why they withdrew from the study.

b. If a participant still does not respond to the email, the same procedure will be attempted twice more. If there is still no contact from the participant by day 4, their support contact (nominated during briefing) will be contacted.

Alongside this, all researchers who undertake briefing (including safety planning) and wellbeing calls will be invited to take part in group supervision sessions once a week during data collection with leading authors. This will be a confidential, safe space to discuss researchers' wellbeing and mental health, with support for any difficulties which may surface.

For peer review only

BMJ Open

Enhancing emotion regulation with an in-situ socially assistive robot among LGBTQ+ youth with self-harm ideation: Protocol for a randomised controlled trial

Journal:	*BMJ Open*
Manuscript ID	bmjopen-2023-079801.R1
Article Type:	Protocol
Date Submitted by the Author:	17-Nov-2023
Complete List of Authors:	Williams, A. Jess; King's College London, Informatics; University of Nottingham, Institute of Mental Health<br>Cleare, Seonaid; University of Glasgow; King's College London, Informatics<br>Borschmann, Rohan; Murdoch Childrens Research Institute; University of Oxford<br>Tench, Christopher; University of Nottingham, Division of Clinical Neuroscience<br>Gross, James; Stanford University<br>Hollis, Chris; University of Nottingham, Division of Psychiatry<br>Chapman-Nisar, Amelia; University of Nottingham<br>Naeche, Nkem; University of Nottingham<br>Townsend, Ellen; University of Nottingham, School of Psychology<br>Slovak, Petr; King's College London; University of Oxford
**Primary Subject Heading**:	Mental health
Secondary Subject Heading:	Mental health
Keywords:	Suicide & self-harm < PSYCHIATRY, MENTAL HEALTH, Adolescents < Adolescent, Sexual and Gender Minorities, Protocols & guidelines < HEALTH SERVICES ADMINISTRATION & MANAGEMENT, Randomized Controlled Trial

SCHOLARONE™
Manuscripts

Enhancing emotion regulation with an in-situ socially assistive robot among LGBTQ+ youth with self-harm ideation: Protocol for a randomised controlled trial

A. Jess Williams, Seonaid Cleare*, Rohan Borschmann, Christopher Tench, James J Gross, Chris Hollis, Amelia Chapman-Nisar, Nkem Naeche, Ellen Townsend, Petr Slovak, on behalf of Digital Youth

*Corresponding author: seonaid.cleare@glasgow.ac.uk
Informatics,
Bush House
Strand Campus
King's College London

Abstract:

Introduction: Purrble, a socially assistive robot, was co-designed with children to support in-situ emotion regulation. Preliminary evidence has found that LGBTQ+ youth are receptive to Purrble and find it to be an acceptable intervention to assist with emotion dysregulation and their experiences of self-harm. The present study is designed to evaluate the impact of access to Purrble among LGBTQ+ youth who have self-harmful thoughts, when compared to waitlist controls.

Methods and analysis: The study is a single-blind, randomised control trial comparing access to the Purrble robot with waitlist control. A total of 168 LGBTQ+ youth aged 16-25 years with current self-harmful ideation will be recruited, all based within the UK.

The primary outcome is emotion dysregulation (Difficulties with Emotion Regulation Scale-8) measured weekly across a 13-week period, including 3 pre-deployment timepoints. Secondary outcomes include self-harm (Self-Harm Questionnaire), anxiety (Generalised Anxiety Disorder-7), and depression (Patient Health Questionnaire-9). We will conduct analyses using linear mixed models to assess primary and secondary hypotheses. Intervention participants will have unlimited access to Purrble over the deployment period, which can be used as much or as little as they like. After all assessments, control participants will receive their Purrble, with all participants keeping the robot after the end of the study.

For peer review only

After the study has ended, a subset of participants will be invited to participate in semi-structured interviews to explore engagement and appropriation of Purrble, considering the young people's own views of Purrble as an intervention device.

Ethics and dissemination: Ethical approval was received from King's College London (RESCM-22/23-34570). Findings will be disseminated in peer review open-access journals and at academic conferences.

Trial registration number: Clinical trial gov: NCT06025942

Strengths and limitations of this study

- This is the first randomised controlled trial to explore the impact of access of Purrble, a socially assistive robot, compared to waitlist control on emotion regulation difficulties within LGBTQ+ youth who have current experiences of self-harmful ideation.
- Purrble was co-designed with youth to support emotion regulation in-situ.
- The study was co-designed with young people who have experience of mental health difficulties (Sprouting Minds), including a detailed safeguarding procedure.
- Participants will not be blinded to participant group due to the nature of the intervention.

Introduction

Self-harm, defined as the intentional poisoning or injury of self, irrespective of intention [1], is a key health concern among sexual orientation and/or gender identity minorities, LGBTQ+ populations [2]. Internationally, young LGBTQ+ people report higher prevalence of self-harmful thoughts and behaviours, anxiety, depression, and substance misuse when compared to their cisgender, heterosexual peers [3-8]. It is well documented that those with a history of self-harm are at greater risk of suicide [9], and recent evidence indicates that LGBTQ+ youth are 3-6 times more likely attempt suicide than their cisgender, heterosexual counterparts [10-11]. Despite the considerable risk of self-harm and associated adverse outcomes [12-13], there is a lack of evidence-based interventions to support LGBTQ+ youth struggling with self-harmful thoughts and behaviours.

Youth who self-harm often do not seek professional help [14-15], and those that do find services (e.g., Child and Adolescent Mental Health Services, Accident and Emergency or social services) to be less helpful sources of support [16] or rarely re-attend services [17]. Factors relating to this can include negative attitudes and behaviours/treatment from health care staff (e.g., withdrawing pain-reducing medication for wound treatment [18]), concerns about confidentiality [19], or perceived stigma surrounding self-harm [20-21]. Among LGBTQ+ people, help-seeking is even more complex, with one in seven avoiding services due to fears of discrimination on the basis of their sexual orientation or gender identity [22].

For peer review only - http://bmjopen.bmj.com/site/about/guidelines.xhtml

Therefore, community-based interventions may be more appropriate to support LGBTQ+ youth engaging in self-harm.

As LGBTQ+ youth are frequent users of digital technologies [23-26], there is an opportunity for digital interventions to support those struggling with self-harm and other mental health difficulties. Evidence suggests that digital interventions support youth to bypass various barriers to help-seeking, such as lack of accessibility, anticipated stigma, inadequate resources, and the desire to be self-reliant [27], which are compounded by unique challenges facing LGBTQ+ youth (e.g., concerns about experiencing stigma or discrimination as a minority [28]).

At present, the field concerning digital interventions among LGBTQ+ youth is small, yet those available are perceived as feasible, acceptable, and relatively effective [29]. However, most focus on physical health such as risk reduction or management of sexually transmitted illnesses (STIs; [29]), with few concerning mental wellbeing [30-33]. These mental health interventions are typically perceived positively by LGBTQ+ youth [30, 32-34], with mixed findings reported by the three studies which considered the impact of the intervention on participants; 1) Rainbow SPARX [32]; 2) an online writing intervention [31]; and 3) QueerViBE [30]. In their pilot trial, Rainbow SPARX (a didactic PC game using Cognitive Behaviour Therapy principles) was associated with large reduction for depressive (d=1.01) and anxious (d=0.95) symptoms [32]. QueerViBE (a series of brief, interactive videos designed for transgender and gender diverse youth) found a moderate decrease in psychological distress (d=0.63) when compared to the control group [30]. However, the expressive writing intervention demonstrated no difference in depressive symptoms in their randomised controlled trial [31]. Therefore, while limited, digital interventions are feasible, acceptable, and potentially effective for improving mental health among LGBTQ+ youth. However, there are currently no evidence-based digital interventions targeting LGBTQ+ youth who struggle with self-harm.

Whilst self-harm among LGBTQ+ youth can be associated with multiple risks, complex experiences, and unique stressors [35-37], a common issue is often emotional dysregulation [38-41]. Experiencing difficulties with emotion regulation (ER) is a well-known transdiagnostic risk factor [42-44], which can be associated with higher risk of self-harm across ages, settings, and genders [45-46]. Typically, LGBTQ+ populations report greater difficulties with ER [40-41], which explains in part the association between LGBTQ+ identity and self-harm [40-41]. Examining how ER can be better supported in young LGBTQ+ people who self-harm through digital intervention may be a helpful preventative strategy to aid LGBTQ+ youth broadly.

To address this, a pilot study was conducted with LGBTQ+ youth who had recent experiences of self-harmful thoughts and/or behaviours using an in-situ, emotion regulation intervention

For peer review only - http://bmjopen.bmj.com/site/about/guidelines.xhtml

device, Purrble [47], designed to provide in-the-moment support, see Intervention section for further details. Purrble was originally developed for children in moments of situated distress, but it has since been well-accepted across child and student populations delivering notable benefits for emotion regulation [47-50]. Among a small sample of 21 LGBTQ+ young people, Purrble was found to be a feasible and acceptable intervention with continued device engagement across a 2-week deployment [47]. Notably, access to Purrble was also associated with a reduction in anxiety symptoms and self-harmful thoughts. Qualitative data indicated that this was linked to Purrble supporting ER practices (e.g., grounding, soothing) to prevent young people acting on their self-harmful urges and, in some cases, preventing them from considering self-harm at all [47]. This is the only study to date, which has explored the impact of a socially assistive robot among LGBTQ+ youth, who are at-risk of self-harm [47]. Based on these findings, and Purrble's original design to support in-situ, bottom-up ER [48-49], it appears that mental health outcomes such as anxiety and self-harm [47, 50] are guided by the proximal change in emotion regulation.

Socially assistive robots (SARs) have previously been used to support children in education [51], family [49] or health settings [52-53], as well as adults with health conditions such as dementia or physical illnesses [54-55]. These studies have shown promising results in the context of motivation, skill development and enhancement, as well as supporting mental health outcomes, e.g., reducing loneliness and stress [49-55]. Similarly, students and at-risk young people have described Purrble robots are a mechanism for comfort and distress relief [47, 50]. However, an ethical challenge raised in SARs literature is the use of these device as a replacement for humans, which could incur negative impacts considering social isolation [56]. Therefore, research utilising SARs should be mindful of this, considering this influence in process analysis, and have additional procedures to prevent overreliance on these devices.

Although, early data relating to Purrble robots is promising [47], there is a lack of robust quantitative data on the impact of the Purrble robot in a wider sample of LGBTQ+ young people who have self-harmed. Evidence is therefore urgently needed to evaluate the efficacy of Purrble in a) delivering measurable changes in ER when compared to a control group, and b) the extent to which this impacts the frequency of self-harmful thoughts and/or anxiety symptoms.

Study objectives

Primary Objective: The primary objective of this study is to evaluate the impact of having access to the Purrble robot, compared with a waitlist control, on emotion regulation difficulties (DERS8) among LGBTQ+ young people with self-harmful thoughts.

Secondary Objectives: The secondary objectives are i) to investigate the impact of having access to Purrble on changes to LGBTQ+ young people's self-harmful thoughts over the trial period, in comparison to a waitlist control group; and ii) to investigate the impact of Purrble

For peer review only - http://bmjopen.bmj.com/site/about/guidelines.xhtml

on changes in symptoms of anxiety (GAD-7), and symptoms of depression (PHQ-9) over the trial period, in comparison to waitlist controls. Finally, this will be the first opportunity to assess whether Purrble remains appealing and helpful to LGBTQ+ youth over an extended period.

Methods

Trial design

The study is a 2-arm randomised controlled trial comparing an intervention group (Purrble robot) with a waitlist control group. The trial period is across 13 weeks, built of 3 pre-deployment assessments and 10 deployment assessments, using weekly, self-reported, validated surveys hosted by Qualtrics (see table 1). The intervention period will commence once Purrble has been deployed to the intervention group, week 4 (T1).

Table 1: Overview of assessment design for both participant groups

**Surveys**	**Pre-deployment (week 1-3)**			**Deployment (week 4-13)**				**Follow-up (week 13+)**
	**T(-2)**	**T(-1)**	**T(0)**	**T1- T4**	**T5**	**T6- T9**	**T10**	
Register interest + screening	X							
Consent	x							
Main assessment	x	x		x		x		
Extended assessment			x		x		x	
Qualitative interviews								x

Analyses will be conducted and reported in accordance with the Consolidated Standards of Reporting Trials (CONSORT; [57-58]), with consideration given to the recommendation of psychological interventions [52]. Outcomes will be assessed 13 times across a 13-week period, including 3 baseline assessments and 10 weeks of deployment, with Purrble being delivered in time for week 4 (T1).

For peer review only

For peer review only - http://bmjopen.bmj.com/site/about/guidelines.xhtml

Intervention

The intervention takes the form of an interactive plush toy-robot (figure 1), which was co-designed with children to support in-the-moment soothing [48-49]. Purrble is framed as an anxious creature, in need of care and attention when it feels distressed. This distress is indicated by a simulated heartbeat using embedded electronics to produce vibration patterns of i) frantic and anxious, to ii) slow, steady, and relaxed. When held, the device emits a frantic heartbeat which can be slowed by stroking movements registered by embedded sensors. Once the device has been "soothed" for long enough, the heartbeat transitions into a purring vibration, indicating a relaxed state. This transition can be achieved in less than 60 seconds but is dependent on the device-human interaction. Further details on the logic model underlying Purrble can be found in supplementary materials 1, or see previous research [49].

[insert figure 1 around here]

Figure 1: Purrble – socially assistive robot

Waitlist – Control

The participants in the control group will be on a waitlist throughout the 13-week trial period. Once data have been collected at the final timepoint (week 13, T10), waitlist participants will receive a Purrble to keep. Waitlist control group was selected following discussions with Sprouting Minds members (see Patient and Public Involvement).

Participants

Eligibility Criteria

When potential participants register their interest for the study, they will be asked demographic questions relating to the eligibility criteria, providing our information for our inclusion criteria. These are: i) being between the ages of 16-25 years (inclusive); ii) identifying as any part of the LGBTQ+ umbrella, iii) having current experiences of self-harmful thoughts (e.g., in the last month), iv) being able to read, write, and speak English, and v) living in the UK for the duration of the study. Exclusion criteria are being outside of the age range, identifying as heterosexual cisgender, not experiencing current self-harmful thoughts, being unable to understand English and living outside the UK.

Recruitment, Randomisation, and Blinding

LGBTQ+ youth will be recruited to the trial by several strategies. These include: i) approaching secondary schools and colleges, ii) social media adverts, iii) advertising through stakeholder charities and organisations (e.g., Bounce Black, Harmless, King's College London

For peer review only - http://bmjopen.bmj.com/site/about/guidelines.xhtml

newsletter), and iv) online platforms (e.g., MQ Participate). Those organisations which involve gatekeepers (e.g., schools, colleges, charities, organisations) will be emailed by one of the leading researchers offering an introductory meeting to discuss the outline of study, explaining safeguarding protocols, and how to share this information with young people. Young LGBTQ+ people will then be able to register their interest in the study, anonymously from gatekeepers. At recruitment, participants will not be blinded to the fact that Purrble was designed to support ER. The information sheet specifies that participants will be asked about mood, self-harm, and ER over the course of the study.

Once eligibility is confirmed and young LGBTQ+ people have provided written informed consent (supplementary material 2), they will be 1:1 randomly assigned to either intervention or waitlist control group, using a computerised algorithm. A stratification procedure will be applied to balance gender identity (transgender and gender diverse youth vs. cisgender) across the two arms. The researcher conducting randomisation will be blind to treatment group allocation. However, the leading researcher and other team members who will be conducting safeguarding will be aware of group allocation. Researchers collecting outcome measures will be blinded to group allocation. Participants will be informed about their assigned condition. The intervention group will receive the Purrble before T1 data are collected, with waitlist participants receiving their Purrble devices after the 3-month follow-up data collection. Participants may withdraw at any time, once they have received their Purrble device it is theirs to keep.

In the event of change of circumstance, such as a serious adverse event, participants are asked to inform the research team. Specifically, for follow-up engagements a standard operation protocol which asks that if a situation changes for a young person (e.g., they are hospitalised) that the young person informs the research team. This will be directly asked ("Has your situation changed at all which might impact how you'd like to engage with the study?") via email, with the understanding that the research team will have time to arrange reasonable adjustments (e.g., if a young person still wants to take part but is within an in-patient service).

Power Analysis

Sample size was determined based on an a-priori power analysis to detect a difference between the two arms when considering the primary outcome measure, the Difficulties with Emotion Regulation Scale-8 (DERS8). On the basis of a pilot with LGBTQ+ youth [47] and other Purrble studies [50, 59], we expect to see a medium effect size for this measure (d=0.4). This would indicate fewer difficulties with ER among young people who had access to Purrble.

With the anticipated medium effect size, simulations were performed involving a range of fixed and random effects. Simulations involved linearly increasing effect over the study period, and sensitivity analysis was performed over a range of scenarios considering the

For peer review only - http://bmjopen.bmj.com/site/about/guidelines.xhtml

slope of effect change over time was either fixed or random. The simulation used a one sided t-test (∝=0.05), and targeted a sample size giving at least 80% power. The statistic to be compared between groups is the change in mean DERS8 score in the 2 weeks preceding intervention (3 assessments in total), to the mean DERS8 assessed at weeks 8, 9, and 10. The averaging over 3 assessments is intended to reduce the known variance in DERS8 when repeatedly assessed [60]. Other simulations considered only comparing the change in DERS8 from baseline (week 0) to week 10, comparing the slope of the effect, assessed using simple linear regression. All simulations suggested better than 60% power with 70 subjects per arm, with the mean change in DERS8 averaged over 3 assessments, and the comparison of slopes, suggesting >80% power. The sample size of 140 is inflated by 20% to account for dropout rate [61], rounding up the total sample to 168.

Outcome Measures

An overview of all measures can be found in Table 2, and full details can be found in supplementary material 3. Primary and mental health measures will be asked at all timepoints, with the Purrble intervention group also receiving two additional engagement measures throughout deployment. An extended survey will replace the weekly survey at three time points, this will include three additional measures to be asked to all participants. All surveys will be distributed via Qualtrics using individualised links for each participant.

These additional measures were selected to explore the association between self-harm and emotion regulation (Process Model Emotion Regulation Questionnaire - PMERQ) [38-41], perceptions of hope (State Hope Scale - SHS) [13], and loneliness (3-item UCLA loneliness scale) [62], to further explore the qualitative findings represented in our pilot study [47]. Our findings have previously indicated that Purrble was used to i) refocus or distract attention during moments of distress by addressing the physical manifestation of their discomfort (PMERQ), and ii) comfort in moments of loneliness and provide self-soothing mechanisms (UCLA, SHS) [47].

Considering participant burden, young people will be informed of the time to complete each weekly survey (15 minutes), the extended survey (22 minutes) and will be compensated for their time.

For peer review only - http://bmjopen.bmj.com/site/about/guidelines.xhtml

289 **Table 2: Summary of the outcome measures**

Outcome Measure	Questions (n)	Type of outcome	Frequency	Scoring	Details of assessment
Primary measure					
Difficulties in emotion regulation scale-8 DERS8; [60]	8	Primary outcome	All timepoints	8-40 (higher = more difficulties)	Difficulties associated with response to situations eliciting negative emotions.
Mental health measures					
Self-Harm Questionnaire screening questions SHQ; [63]	3	Mental health	All timepoints	Analysed separately	Frequency and risk of self-harm thoughts, suicidal ideation, and behaviour.
Self-Harm Questionnaire additional items SHQ; [63]	22	Covariate	Once (baseline)	Analysed separately	4 dimensions of self-harm (NSSI, suicide attempts, suicide threats, suicide ideation).
Generalised Anxiety Disorder questionnaire GAD-7; [64]	7	Mental health	All timepoints	0-21 (higher = greater severity)	Presence and severity of generalized anxiety disorder
Patient Health Questionnaire PHQ-9; [65]	9	Mental health	All timepoints	0-27 (higher = greater severity)	Severity of depressive symptoms
Proximal & mechanistic measures					
Process Model of Emotion Regulation Questionnaire PMERQ; [66]	9	Mechanistic	3 timepoints	2 subscales Average across each subscale, higher = greater endorsement.	Attentional deployment subscales; focus on engagement and disengagement.
State Hope Scale SHS; [67]	6	Proximal	3 timepoints	6-48 (higher = greater state hopefulness)	Goal-directed thinking; agency and pathways.
UCLA Loneliness scale for children; [68]	3	Proximal	3 timepoints	3-12 (higher = more loneliness)	Subjective feelings of loneliness.

For peer review only - http://bmjopen.bmj.com/site/about/guidelines.xhtml

Engagement measures					
Bespoke Purrble questions; [59]	7	Engagement	Deployment	Analysed separately	Purrble use and perceived usefulness.
Twente Engagement With eHealth Technologies Scale TWEETS; [69]	9	Engagement	Deployment	3 subscales Total score per subscale = greater engagement	Engagement with intervention device; behavioural, cognitive, and affective engagements.

Post deployment Interviews

We will collect semi-structured interview data from LGBTQ+ young people from up to 40% of the intervention group (n=37), and approximately 20% of the control group (n=17). The interviews will be conducted following the deployment period. We will specifically aim to recruit young people who demonstrated the highest and lowest changes in the outcome data over the trial to explore and understand the potential moderators relating to the intervention and mental health across the trial period.

The semi-structured interview will explore the engagement and appropriation of the Purrble device, whether LGBTQ+ young people had felt that this had helped them with their ER, mental health more broadly, or self-harmful thoughts, and how Purrble may (or may not) be suitable for other audiences. We will compare these experiences between intervention and control participants, exploring other mechanisms used by LGBTQ+ youth who experience self-harm.

Hypotheses

Primary hypothesis

Across the trial, we hypothesise that access to the Purrble intervention (compared to the waitlist control) will lead to a direct decrease in self-reported difficulties with emotion regulation as measured by the primary outcome (DERS8), averaged between three pre-deployment (weeks 1-3) and our final three deployment assessments (weeks 11-13).

Secondary hypothesis

Intervention effects will be moderated by engagement with the device, measured by bespoke questions and the TWEETS questionnaire. Secondary outcomes in the Purrble effectiveness trial are: self-harmful thoughts, symptoms of anxiety, and symptoms of

For peer review only

depression. These three constructs were selected as secondary outcomes based on the high prevalence of these experiences among LGBTQ+ youth [3-8] and their association with poor ER [38-41]. The three secondary hypotheses are as listed when compared to waitlist controls:

1. Engagement with the Purrble intervention will reduce the frequency of self-reported self-harmful thoughts (SHQ).
2. Engagement with the Purrble intervention will reduce the severity of self-reported anxiety symptoms (GAD-7).
3. Engagement with the Purrble intervention will reduce the severity of self-reported depression symptoms (PHQ-9).

Additional analyses

Additional hypotheses aim to understand the impact of Purrble on relevant proximal and mechanistic outcomes. The following hypotheses will be investigated across the trial:

1. Greater within-group changes will be seen among intervention group participants, with increasing levels of endorsement for attentional deployment (PMERQ), than among those participants of the control group.
2. There will be a greater increase in state hopefulness (SHS) in the Purrble intervention group than the waitlist control.
3. Participants in the Purrble intervention group will report lower loneliness (UCLA) than those in the waitlist control group.

Statistical analyses

Testing the hypothesis that access to the Purrble intervention will lead to a reduction in emotion dysregulation, as measured by the composite primary outcome, will be done using a one-sided t-test.

As exploratory analyses linear mixed models will be fitted to gain insight into how emotion dysregulation is altered with access to the intervention. In particular, we will regress the weekly outcome score on an indicator for the Purrble condition, a linear time trend, and an interaction between the treatment indicator and time to examine differential trends in the two groups. We will adjust for baseline covariates and include participant-level random intercepts and slopes to account for persistent baseline differences between young people as well as person-specific time trends in the outcome. While the outcome is limited to DERS8 scores ranging from 8-40, we will model it as continuous data.

For secondary aims, we will use analogous linear mixed models to assess the impact of Purrble on relevant outcomes (cf., hypotheses above), adding baseline DERS8 as another covariate. We will not adjust for multiple comparisons, as these are exploratory aims meant to be hypothesis-generating. Similarly, we will also assess the impact of access to Purrble on

For peer review only

changes in proximal outcomes, as well as explore whether these appear to moderate changes on primary and secondary outcomes.

Patient and Public Involvement

The study design was discussed with, and approved by, Sprouting Minds members (MRC Digital Youth Young Person Advisory group), with specific input considering the intervention arms and safeguarding procedures. These young people highlighted that "waitlist control" conditions mimic clinical experiences of waiting for services, therefore this was considered an acceptable and realistic control. Whereas safeguarding procedures needed to balance participant autonomy and ensuring safe research.

Ethics and dissemination

This manuscript has been written with insights from the Spirit 2013 checklist (supplementary material 4). The study will be conducted according to local regulations and the Declaration of Helsinki of 1975, revised in 2008. The Ethical Committee at King's College London, UK approved the study (RESCM-22/23-34570). Written consent will be obtained from all participants prior to commencing their involvement in the study, with explicit understanding of the study and safeguarding procedures (see supplementary materials 5) being obtained during study briefing sessions. The trial is registered with ClinicalTrivals.gov (NCT06025942).

We aim for our findings (and any modifications to this protocol) to be disseminated across academic fields (human-computer interactions, psychology, implementation sciences), alongside showcasing the findings to LGBTQ+ youth, community groups, and wider stakeholders. This will be achieved through presentations at national and international conferences, peer-reviewed journal publications, community outreach and PPI events. During dissemination, we will be liaising with youth populations to establish next steps for this research, considering additional co-design of materials to sit around/alongside Purrble.

Acknowledgements

The authors (AJW, ET, NN, ACN, CH, PS) acknowledge the support of the UK Research and Innovation (UKRI) Digital Youth Programme award [MRC project reference MR/W002450/1] which is part of the AHRC/ESRC/MRC Adolescence, Mental Health and the Developing Mind programme. The authors would like to thank our PPI group Sprouting Minds, including the two co-chairs, Sarah Doherty and Lucy-Paige Willingham, for their ongoing support and contributions to the project. RB receives salary and research support from an Australian National Health and Medical Research Council (NHMRC) Emerging Leadership Investigator Grant (EL2; GNT2008073).
We would also like to thank Mathijs Lucassen for his input while developing this trial.

For peer review only - http://bmjopen.bmj.com/site/about/guidelines.xhtml

Author contributions

AJW, ET, PS conceived the study. AJW and PS designed the study, with statistical expertise from CT and patient and public input from NN and ACN. AJW designed the study materials, which were reviewed with feedback between NN, ACN, and SC. AJW obtained ethical approval. JG, ET, PS and AJW contributed to measure selection. AJW drafted the first version of the protocol manuscript for publication, with input from all authors (AJW, RB, SC, ET, NN, ACN, CH, PS). RB and AJW revised the submitted protocol manuscript together.

Funding

This study is funded by the UKRI MRC Digital Youth (MR/W002450/1) and the UKRI Future Leaders Fellowship (MR/T041897/1). Funders have no involvement with the study design, data collection, management, analysis, interpretation or dissemination. This is now included.

Competing interests statement

PS has been involved in the development of what is now Purrble as part of his postdoctoral fellowship; and serves as a paid research adviser to the Committee for Children but has no financial stake in either CfC (Purrble brand owners) or Sproutel (company manufacturing Purrbles). Neither Committee for Children nor Sproutel had access to the data or had been part of the data collection in any way, nor did they approve or see the publication before it was submitted. There were no conflicting interests among the remaining research team.

Data availability statement

Data will be available upon reasonable request once the trial is complete. To date there is no data that has been collected or analysed for this study. The data will be held at King's College London, following the data management agreement of Digital Youth.

References:

1. Kendall T, Taylor C, Bhatti H, Chan M, Kapur N. Longer term management of self harm: summary of NICE guidance. Bmj. 2011 Nov 23;343. doi.org/10.1136/bmj.d7073
2. World Health Organisation. Suicide [fact sheet] [Internet]. World Health Organization; 2021 [cited 2023 Mar 24]. Available from: https://www.who.int/news-room/fact-sheets/detail/suicide
3. Coker TR, Austin SB, Schuster MA. The health and health care of lesbian, gay, and bisexual adolescents. Annual review of public health. 2010 Apr 21;31:457-77. doi.org/10.1146/annurev.publhealth.012809.103636
4. Haas AP, Eliason M, Mays VM, Mathy RM, Cochran SD, D'Augelli AR, Silverman MM, Fisher PW, Hughes T, Rosario M, Russell ST. Suicide and suicide risk in lesbian, gay,

For peer review only - http://bmjopen.bmj.com/site/about/guidelines.xhtml

bisexual, and transgender populations: Review and recommendations. Journal of homosexuality. 2010 Dec 30;58(1):10-51. DOI: 10.1080/00918369.2011.534038
5. Marshal MP, Dietz LJ, Friedman MS, Stall R, Smith HA, McGinley J, Thoma BC, Murray PJ, D'Augelli AR, Brent DA. Suicidality and depression disparities between sexual minority and heterosexual youth: A meta-analytic review. Journal of adolescent health. 2011 Aug 1;49(2):115-23. doi.org/10.1016/j.jadohealth.2011.02.005
6. Saewyc EM. Research on adolescent sexual orientation: Development, health disparities, stigma, and resilience. Journal of research on adolescence. 2011 Mar;21(1):256-72. doi.org/10.1111/j.1532-7795.2010.00727.x
7. McDermott E, Hughes E, Rawlings V. Norms and normalisation: understanding lesbian, gay, bisexual, transgender and queer youth, suicidality and help-seeking. Culture, Health & Sexuality. 2017 Feb 1;20(2):156-72. doi.org/10.1080/13691058.2017.1335435
8. Irish M, Solmi F, Mars B, King M, Lewis G, Pearson RM, Pitman A, Rowe S, Srinivasan R, Lewis G. Depression and self-harm from adolescence to young adulthood in sexual minorities compared with heterosexuals in the UK: a population-based cohort study. The Lancet Child & Adolescent Health. 2019 Feb 1;3(2):91-8. doi.org/10.1016/S2352-4642(18)30343-2
9. Chan MK, Bhatti H, Meader N, Stockton S, Evans J, O'Connor RC, Kapur N, Kendall T. Predicting suicide following self-harm: systematic review of risk factors and risk scales. The British Journal of Psychiatry. 2016 Oct;209(4):277-83. doi: 10.1192/bjp.bp.115.170050
10. Di Giacomo E, Krausz M, Colmegna F, Aspesi F, Clerici M. Estimating the risk of attempted suicide among sexual minority youths: a systematic review and meta-analysis. JAMA pediatrics. 2018 Dec 1;172(12):1145-52. doi:10.1001/jamapediatrics.2018.2731
11. Marchi M, Arcolin E, Fiore G, Travascio A, Uberti D, Amaddeo F, Converti M, Fiorillo A, Mirandola M, Pinna F, Ventriglio A. Self-harm and suicidality among LGBTIQ people: a systematic review and meta-analysis. International review of psychiatry. 2022 May 19;34(3-4):240-56. doi.org/10.1080/09540261.2022.2053070
12. Warne N, Heron J, Mars B, Solmi F, Biddle L, Gunnell D, Hammerton G, Moran P, Munafò M, Penton-Voak I, Skinner A. Emotional dysregulation in childhood and disordered eating and self-harm in adolescence: prospective associations and mediating pathways. Journal of child psychology and psychiatry. 2023 May;64(5):797-806. doi.org/10.1111/jcpp.13738
13. Mars B, Heron J, Klonsky ED, Moran P, O'Connor RC, Tilling K, Wilkinson P, Gunnell D. Predictors of future suicide attempt among adolescents with suicidal thoughts or non-suicidal self-harm: a population-based birth cohort study. The Lancet Psychiatry. 2019 Apr 1;6(4):327-37. doi.org/10.1016/S2215-0366(19)30030-6
14. Geulayov G, Casey D, McDonald KC, Foster P, Pritchard K, Wells C, Clements C, Kapur N, Ness J, Waters K, Hawton K. Incidence of suicide, hospital-presenting non-fatal self-harm, and community-occurring non-fatal self-harm in adolescents in England (the iceberg model of self-harm): a retrospective study. The Lancet Psychiatry. 2018 Feb 1;5(2):167-74. doi.org/10.1016/S2215-0366(17)30478-9

For peer review only - http://bmjopen.bmj.com/site/about/guidelines.xhtml

15. Geulayov G, Borschmann R, Mansfield KL, Hawton K, Moran P, Fazel M. Utilization and acceptability of formal and informal support for adolescents following self-harm before and during the first COVID-19 lockdown: results from a large-scale English schools survey. Frontiers in psychiatry. 2022 Jun 24;13:881248. doi.org/10.3389/fpsyt.2022.881248
16. Holland J, Sayal K, Berry A, Sawyer C, Majumder P, Vostanis P, Armstrong M, Harroe C, Clarke D, Townsend E. What do young people who self-harm find helpful? A comparative study of young people with and without experience of being looked after in care. Child and adolescent mental health. 2020 Sep;25(3):157-64. Doi.org/10.1111/camh.12384
17. Michelmore L, Hindley P. Help-seeking for suicidal thoughts and self-harm in young people: A systematic review. Suicide and Life-Threatening Behavior. 2012 Oct;42(5):507-24. doi.org/10.1111/j.1943-278X.2012.00108.x
18. Owens C, Hansford L, Sharkey S, Ford T. Needs and fears of young people presenting at accident and emergency department following an act of self-harm: secondary analysis of qualitative data. The British Journal of Psychiatry. 2016 Mar;208(3):286-91. doi.org/10.1192/bjp.bp.113.141242
19. Williams AJ, Nielsen E, Coulson NS. "They aren't all like that": Perceptions of clinical services, as told by self-harm online communities. Journal of health psychology. 2020 Nov;25(13-14):2164-77. doi.org/10.1177/13591053187884
20. Long M, Manktelow R, Tracey A. We are all in this together: working towards a holistic understanding of self-harm. Journal of Psychiatric and Mental Health Nursing. 2013 Mar;20(2):105-13. doi.org/10.1111/j.1365-2850.2012.01893.x
21. Bathje G, Pryor J. The relationships of public and self-stigma to seeking mental health services. Journal of Mental Health Counseling. 2011 Apr 1;33(2):161-76. doi.org/10.17744/mehc.33.2.g6320392741604l1
22. Stonewall. LGBT in Britain Health Report [Internet]. Stonewall; 2018 [cited 2023 Mar 24]. Available from: https://www.stonewall.org.uk/system/files/lgbt_in_britain_health.pdf
23. Steinke J, Root-Bowman M, Estabrook S, Levine DS, Kantor LM. Meeting the needs of sexual and gender minority youth: formative research on potential digital health interventions. Journal of Adolescent Health. 2017 May 1;60(5):541-8. doi.org/10.1016/j.jadohealth.2016.11.023
24. Bowen D, Jabson J, Kamen C. mHealth: an avenue for promoting health among sexual and gender minority populations?. Mhealth. 2016;2. doi: 10.21037/mhealth.2016.09.01
25. Strauss P, Morgan H, Toussaint DW, Lin A, Winter S, Perry Y. Trans and gender diverse young people's attitudes towards game-based digital mental health interventions: A qualitative investigation. Internet Interventions. 2019 Dec 1;18:100280. Doi.org/10.1016/j.invent.2019.100280
26. Craig SL, McInroy L. You can form a part of yourself online: The influence of new media on identity development and coming out for LGBTQ youth. Journal of Gay & Lesbian Mental Health. 2014 Jan 1;18(1):95-109. doi.org/10.1080/19359705.2013.777007

For peer review only - http://bmjopen.bmj.com/site/about/guidelines.xhtml

27. Gulliver A, Griffiths KM, Christensen H. Perceived barriers and facilitators to mental health help-seeking in young people: a systematic review. BMC psychiatry. 2010 Dec;10(1):1-9. doi.org/10.1186/1471-244X-10-113
28. McIntyre J, Daley A, Rutherford K, Ross LE. Systems-level barriers in accessing supportive mental health services for sexual and gender minorities: Insights from the provider's perspective. Canadian Journal of Community Mental Health. 2012 Mar 22;30(2):173-86. doi.org/10.7870/cjcmh-2011-0023
29. Gilbey D, Morgan H, Lin A, Perry Y. Effectiveness, acceptability, and feasibility of digital health interventions for LGBTIQ+ young people: systematic review. Journal of medical Internet research. 2020 Dec 3;22(12):e20158. doi:10.2196/20158
30. Martin S. *Developing and evaluating QueerViBE: an online intervention to empower trans and non-binary youth* (Doctoral dissertation, Anglia Ruskin University). [cited 2023 Mar 24].
31. Pachankis JE, Goldfried MR. Expressive writing for gay-related stress: psychosocial benefits and mechanisms underlying improvement. Journal of consulting and clinical psychology. 2010 Feb;78(1):98. Doi.org/10.1037/a0017580
32. Lucassen MF, Merry SN, Hatcher S, Frampton CM. Rainbow SPARX: A novel approach to addressing depression in sexual minority youth. Cognitive and Behavioral Practice. 2015 May 1;22(2):203-16. Oi.org/10.1016/j.cbpra.2013.12.008
33. Fleming JB, Hill YN, Burns MN. Usability of a culturally informed mHealth intervention for symptoms of anxiety and depression: feedback from young sexual minority men. JMIR human factors. 2017 Aug 25;4(3):e7392. doi: 10.2196/humanfactors.7392
34. Fish JN, Williams ND, McInroy LB, Paceley MS, Edsall RN, Devadas J, Henderson SB, Levine DS. Q Chat Space: assessing the feasibility and acceptability of an internet-based support program for LGBTQ youth. Prevention science. 2021 Sep 6:1-2. doi.org/10.1007/s11121-021-01291-y
35. Williams AJ, Arcelus J, Townsend E, Michail M. Feasibility and acceptability of experience sampling among LGBTQ+ young people with self-harmful thoughts and behaviours. Frontiers in psychiatry. 2022 Aug 17;13:916164. Doi.org/10.3389/fpsyt.2022.916164
36. Williams AJ, Arcelus J, Townsend E, Michail M. Understanding the processes underlying self-harm ideation and behaviors within LGBTQ+ young people: A qualitative study. Archives of suicide research. 2021 Apr 3;27(2):380-96. doi.org/10.1080/13811118.2021.2003273
37. Hatchel T, Polanin JR, Espelage DL. Suicidal thoughts and behaviors among LGBTQ youth: Meta-analyses and a systematic review. Archives of suicide research. 2019 Jan 2;25(1):1-37. doi.org/10.1080/13811118.2019.1663329
38. Chen WL, Chun CC. Association between emotion dysregulation and distinct groups of non-suicidal self-injury in Taiwanese female adolescents. International journal of environmental research and public health. 2019 Sep;16(18):3361. Doi.org/10.3390/ijerph16183361
39. Clapham R, Brausch A. Internalizing and externalizing symptoms moderate the relationship between emotion dysregulation and suicide ideation in adolescents.

For peer review only

Child Psychiatry & Human Development. 2022 Sep 6:1-2. doi.org/10.1007/s10578-022-01413-9

40. Kapatais A, Williams AJ, Townsend E. The mediating role of emotion regulation on self-harm among gender identity and sexual orientation minority (LGBTQ+) individuals. Archives of suicide research. 2023 Apr 3;27(2):165-78. doi.org/10.1080/13811118.2022.2064254
41. Fraser G, Wilson MS, Garisch JA, Robinson K, Brocklesby M, Kingi T, O'Connell A, Russell L. Non-suicidal self-injury, sexuality concerns, and emotion regulation among sexually diverse adolescents: A multiple mediation analysis. Archives of suicide research. 2018 Jul 3;22(3):432-52. doi.org/10.1080/13811118.2017.1358224
42. Sloan E, Hall K, Moulding R, Bryce S, Mildred H, Staiger PK. Emotion regulation as a transdiagnostic treatment construct across anxiety, depression, substance, eating and borderline personality disorders: A systematic review. Clinical psychology review. 2017 Nov 1;57:141-63. Doi.org/10.1016/j.cpr.2017.09.002
43. Gross JJ. Emotion regulation: Current status and future prospects. Psychological inquiry. 2015 Jan 2;26(1):1-26. doi.org/10.1080/1047840X.2014.940781
44. Fernandez KC, Jazaieri H, Gross JJ. Emotion regulation: A transdiagnostic perspective on a new RDoC domain. Cognitive therapy and research. 2016 Jun;40:426-40. doi.org/10.1007/s10608-016-9772-2
45. Wolff JC, Thompson E, Thomas SA, Nesi J, Bettis AH, Ransford B, Scopelliti K, Frazier EA, Liu RT. Emotion dysregulation and non-suicidal self-injury: A systematic review and meta-analysis. European Psychiatry. 2019 Jun;59:25-36. Doi.org/10.1016/j.eurpsy.2019.03.004
46. Fox KR, Franklin JC, Ribeiro JD, Kleiman EM, Bentley KH, Nock MK. Meta-analysis of risk factors for nonsuicidal self-injury. Clinical psychology review. 2015 Dec 1;42:156-67. Doi.org/10.1016/j.cpr.2015.09.002
47. Williams, AJ, Townsend, T, Naeche, N, Chapman-Nisar, A, Hollis, C, Slovak, P. Investigating the feasibility, acceptability and appropriation of a socially assistive robot among minority youth at-risk of self-harm. [in prep]
48. Slovák P, Theofanopoulou N, Cecchet A, Cottrell P, Altarriba Bertran F, Dagan E, Childs J, Isbister K. " I just let him cry... Designing Socio-Technical Interventions in Families to Prevent Mental Health Disorders. Proceedings of the ACM on Human-Computer Interaction. 2018 Nov 1;2(CSCW):1-34. Doi.org/10.1145/3274429
49. Theofanopoulou N, Isbister K, Edbrooke-Childs J, Slovák P. A smart toy intervention to promote emotion regulation in middle childhood: Feasibility study. JMIR mental health. 2019 Aug 5;6(8):e14029. doi: 10.2196/14029
50. Williams AJ, Freed M, Theofanopoulou N, Roquet CD, Klasnja P, Gross JJ, Schleider J, Slovak P. Feasibility, perceived impact, and acceptability of a socially assistive robot to support emotion regulation with highly anxious university students: an open trial. JMIR Mental Health. 2023. [in prep]
51. Papadopoulos I, Lazzarino R, Miah S, Weaver T, Thomas B, Koulouglioti C. A systematic review of the literature regarding socially assistive robots in pre-tertiary education. Computers & Education. 2020 Oct 1;155:103924. doi.org/10.1016/j.compedu.2020.103924

For peer review only

For peer review only - http://bmjopen.bmj.com/site/about/guidelines.xhtml

52. Crossman MK, Kazdin AE, Kitt ER. The influence of a socially assistive robot on mood, anxiety, and arousal in children. Professional Psychology: Research and Practice. 2018 Feb;49(1):48. doi.org/10.1037/pro0000177
53. Kabacińska K, Prescott TJ, Robillard JM. Socially assistive robots as mental health interventions for children: A scoping review. International Journal of Social Robotics. 2021 Aug;13:919-35. doi.org/10.1007/s12369-020-00679-0
54. Šabanović S, Chang WL, Bennett CC, Piatt JA, Hakken D. A robot of my own: participatory design of socially assistive robots for independently living older adults diagnosed with depression. InHuman Aspects of IT for the Aged Population. Design for Aging: First International Conference, ITAP 2015, Held as Part of HCI International 2015, Los Angeles, CA, USA, August 2-7, 2015. Proceedings, Part I 1 2015 (pp. 104-114). Springer International Publishing. doi.org/10.1007/978-3-319-20892-3_11
55. Koh WQ, Ang FX, Casey D. Impacts of low-cost robotic pets for older adults and people with dementia: scoping review. JMIR rehabilitation and assistive technologies. 2021 Feb 12;8(1):e25340. doi: 10.2196/25340
56. Koh WQ, Vandemeulebroucke T, Gastmans C, Miranda R, Van den Block L. The ethics of pet robots in dementia care settings: Care professionals' and organisational leaders' ethical intuitions. Frontiers in psychiatry. 2023 Jan 23;14:1052889. doi.org/10.3389/fpsyt.2023.1052889
57. Schulz KF, Altman DG, Moher D, CONSORT Group*. CONSORT 2010 statement: updated guidelines for reporting parallel group randomized trials. Annals of internal medicine. 2010 Jun 1;152(11):726-32. doi.org/10.7326/0003-4819-152-11-201006010-00232
58. Montgomery P, Grant S, Mayo-Wilson E, Macdonald G, Michie S, Hopewell S, Moher D. Reporting randomised trials of social and psychological interventions: the CONSORT-SPI 2018 Extension. Trials. 2018 Dec;19(1):1-4. doi.org/10.1186/s13063-018-2733-1
59. Daudén Roquet C, Theofanopoulou N, Freeman JL, Schleider J, Gross JJ, Davis K, Townsend E, Slovak P. Exploring Situated & Embodied Support for Youth's Mental Health: Design Opportunities for Interactive Tangible Device. In Proceedings of the 2022 CHI Conference on Human Factors in Computing Systems 2022 Apr 29 (pp. 1-16). doi.org/10.1145/3491102.3502135
60. Penner F, Steinberg L, Sharp C. The Development and Validation of the Difficulties in Emotion Regulation Scale-8: Providing Respondents with a Uniform Context That Elicits Thinking About Situations Requiring Emotion Regulation. Journal of Personality Assessment. 2022 Oct 7:1-0. Doi.org/10.1080/00223891.2022.2133722
61. Cohen KA, Schleider JL. Adolescent dropout from brief digital mental health interventions within and beyond randomized trials. Internet Interventions. 2022 Mar 1;27:100496. Doi.org/10.1016/j.invent.2022.100496
62. Shaw RJ, Cullen B, Graham N, Lyall DM, Mackay D, Okolie C, Pearsall R, Ward J, John A, Smith DJ. Living alone, loneliness and lack of emotional support as predictors of suicide and self-harm: A nine-year follow up of the UK Biobank cohort. Journal of Affective Disorders. 2021 Jan 15;279:316-23. Doi.org/10.1016/j.jad.2020.10.026

For peer review only - http://bmjopen.bmj.com/site/about/guidelines.xhtml

63. Ougrin D, Boege I. Brief report: the Self Harm Questionnaire: a new tool designed to improve identification of self harm in adolescents. Journal of adolescence. 2013 Feb 1;36(1):221-5. Doi.org/10.1016/j.adolescence.2012.09.006
64. Spitzer RL, Kroenke K, Williams JB, Löwe B. A brief measure for assessing generalized anxiety disorder: the GAD-7. Archives of internal medicine. 2006 May 22;166(10):1092-7. doi:10.1001/archinte.166.10.1092
65. Kroenke K, Spitzer RL, Williams JB. The PHQ-9: validity of a brief depression severity measure. Journal of general internal medicine. 2001 Sep;16(9):606-13. doi.org/10.1046/j.1525-1497.2001.016009606.x
66. Olderbak S, Uusberg A, MacCann C, Pollak KM, Gross JJ. The Process Model of Emotion Regulation Questionnaire: assessing individual differences in strategy stage and orientation. Assessment. 2022 Dec 9:10731911221134601.
67. Snyder CR, Sympson SC, Ybasco FC, Borders TF, Babyak MA, Higgins RL. Development and validation of the State Hope Scale. Journal of personality and social psychology. 1996 Feb;70(2):321. doi.org/10.1037/0022-3514.70.2.321
68. Testing of loneliness questions in surveys - Office for National Statistics [Internet]. Ons.gov.uk. 2018. Available from: https://www.ons.gov.uk/peoplepopulationandcommunity/wellbeing/compendium/nationalmeasurementofloneliness/2018/testingoflonelinessquestionsinsurveys. [cited 2023 Mar 24].
69. Kelders SM, Kip H, Greeff J. Psychometric evaluation of the TWente Engagement with Ehealth Technologies Scale (TWEETS): evaluation study. Journal of medical internet research. 2020 Oct 9;22(10):e17757. doi: 10.2196/17757

For peer review only

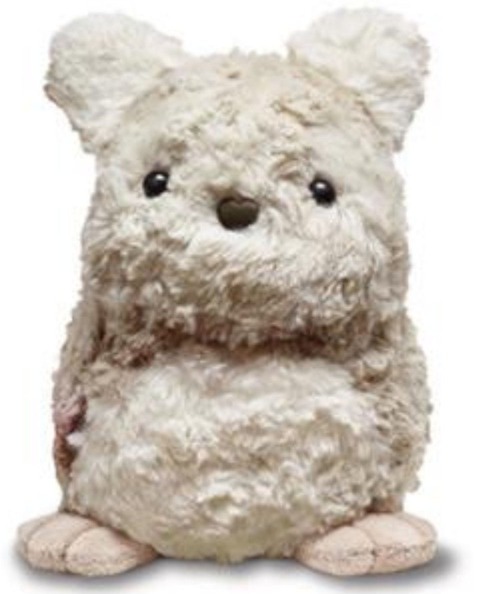

Figure 1: Purrble – socially assistive robot

For peer review only

Supplementary material 1

Purrble logic model

Purrble operates on 3 levels:

1. Purrble directly provides in-the-moment soothing support in naturally occurring emotional moments when one would attempt to calm down. The physical and interactive design aims to tap into known regulatory factors using the extended Process Model of Emotion Regulation (Gross, 2015). This focuses on two separate stages of the ER process; i) the attentional deployment stage (Aldao et al., 2015; Sheppes & Gross, 2012; Sheppes et al., 2014; Farb et al., 2014) by shifting attention from the emotional situation towards interacting with the device, and ii) the response modulation stage (Beetz et al., 2012; Dore et al., 2017; Reeck et al., 2016; Coan et al., 2006; Crossman et al., 2018; Rabbit et al., 2015) by facilitating down-regulation through pleasant tactical interactions simulating the emotion regulatory effect of human-animal interactions.

2. Mechanisms of Purrble are designed to facilitate long-term engagement, by building on positive subjective experiences of in-the-moment soothing. As the device is framed as an anxious creature needing to be cared for, the key driver is that interactions are framed as helping regulate others' emotions (Dore et al., 2017; Cosley et al., 2010; Taylor, 2011), alongside facilitating a sense of relationship and responsibility for the well-being of the creature (Turkle, 2007; Hayashi & Kato, 2016; Donath, 2004; Lee et al., 2010).

3. Through repeated, soothing, and positive interactions with Purrble over time, it is anticipated that there will be a shift in ER practices and implicit beliefs about emotions (Ford & Gross, 2018), specifically the controllability of one's emotions (Schleider & Weisz, 2016).

For peer review only

Supplementary material 2

CONSENT FORM FOR PARTICIPANTS IN RESEARCH PROJECTS

Please complete this form after you have read the Information Sheet and/or listened to an explanation about the research

**Title of project:** An exploratory investigation of the acceptability and feasibility of using an in-situ socially assistive robot with at-risk young people.	
**Ethical review reference number: 34570**	**Version number: 3-23**

Please read and confirm your consent to taking part in this project by initialing all boxes, and signing (by typing your name) below:	Tick or initial
1. I confirm that I have read and understood the information sheet dated **3-23** for the above project. I have had the opportunity to consider the information and asked questions which have been answered to my satisfaction.	
2. I consent voluntarily to be a participant in this project and understand that I can refuse to take part and can withdraw from the project at any time, without having to give a reason, up to 2 weeks following my completion of the study.	
3. I consent to the processing of my personal information for the purposes explained to me in the Information Sheet. I understand that such information will be handled under the terms of UK data protection law, including the UK General Data Protection Regulation (UK GDPR) and the Data Protection Act 2018.	
4. I understand that my anonymised information may be subject to review by responsible individuals from the College for monitoring and audit purposes.	
5. I understand that confidentiality and anonymity will be maintained, and it will not be possible to identify me in any research outputs.	
6. I agree that the researcher/research team may use my data for future research and understand that any such use of identifiable data would be reviewed and approved by a research ethics committee. (In such cases, as with this project, data would not be identifiable in any report).	
7. I confirm that I am between 16-25 years old, who currently lives in the UK and identifies as any part of the LGBTQ+ umbrella.	
8. I understand that I need to provide the name and email address for my support contact (who will only be contacted to i) inform them that you are taking part in a mental health study, and ii) in the event that you can not be reached for a wellbeing check after 3 daily contact attempts via phone/email).	
9. I understand I will be randomised into either an intervention or control group, which impacts when I receive Purrble but irrespective I will be paid for all surveys completed across the 13 week period.	
10. I consent to completing three baseline surveys at the start of the study (weeks 1-3).	

For peer review only

11. I consent to be contacted via SMS to complete a weekly survey for 13 weeks (including the three baseline surveys).	
12. I consent to be invited to take part in an online interview to discuss my experience of the study.	
13. I consent to my interview being audio recorded, and shared with a third-party transcriber who will have signed a confidentiality agreement.	
14. I consent to be invited to take part in a co-designed workshop following the study.	
15. I confirm that I am happy to share my contact information as part of the study (name, email, phone number, home address)	
16. I understand that if I take part in the study, I will receive a Purrble through Royal Mail to my home address.	
17. I agree to take part in this project.	

Name:

Email address:

Your phone number:

Home address:

Name of Participant **Date** **Signature**

Name of Researcher **Date** **Signature**

If you have any questions or require further information, please contact

Researcher: A. Jess Williams

Email: amy_jess.williams@kcl.ac.uk

PI: Petr Slovak

Email: petr.slovak@kcl.ac.uk

For peer review only

Supplementary material 3

Full Measurement Details

Primary Outcome Measure

The primary outcome measure in this study is the Difficulties with Emotion Regulation Scale-8 (DERS8) [60]. This will be given to participants as part of the main assessment at all time-points. The DERS8 contains 8 items which represent affect, thoughts, and actions in response to situations which elicit negative emotions. A single total score is calculated from all items, with higher scores indicating more difficulties with emotion.

Mental Health Measures

Self-harmful thoughts and behaviours will be assessed using the Self-Harm Questionnaire (SHQ) [63]. At baseline, those who indicate experiences of self-harm behaviour will be invited to complete the second section about historical self-harm behaviour. At all other timepoints, only the three screening questions will be presented; these query the frequency of self-harmful thoughts, suicidal ideation, and self-harmful behaviour. The items are scored individually from “no thoughts/behaviour” to “yes, five or more times”. The wording for these items will be adapted from “have you ever…” to “in the last week/month” (depending on assessment point).

Anxiety symptoms will be assessed using the Generalised Anxiety Disorder-7 (GAD-7) [64]. This is a seven-item instrument, used to identify or assess the severity of generalised anxiety disorder. Each item asks the individual to rate the severity of their symptoms over the time period. The total score is calculated by summing all items and ranges from 0-21. Higher scores indicate more severe levels of anxiety symptoms. The wording for these items will be adapted from “over the last two weeks” to “in the last week/month” (depending on assessment point).

Depressive symptoms will be assessed using the Patient Health Questionnaire-9 (PHQ9) [65]. Across nine items, this instrument measures the severity of depressive symptoms, the total score being calculated by summing all items with responses ranging from 0-27. Higher scores indicate greater depressive severity. Again, the wording for these items will be adapted from “over the last two weeks” to “in the last week/month” (depending on assessment point).

Proximal & Mechanistic Measures

All proximal and mechanistic measures will only be asked at three timepoints (T0, T5, T10), this is to reduce participant burden and encourage engagement, while also obtaining exploratory data to understand the impact of access to Purrble in greater detail. All additional measures total to 18 extra items, adding ~7 minutes to the assessment.

The Process Model of Emotion Regulation Questionnaire [66] is a 45-item measure which considers 10 ER strategies across the five stages of the Process Model of ER, and particularly how these strategies are used to decrease negative emotions. We will include 2 subscales

For peer review only

focusing on attention deployment (focusing elsewhere (4 items) and cognitive distraction (5 items)). Each subscale is scored by taking the average of item-level responses.

Hopefulness will be assessed using the State Hope Scale [67]. This is a six-item instrument concerning ongoing goal-directed thinking (agency and pathways). The total score (6-48) is calculated by summing responses, with higher scores relating to greater state hopefulness.

Loneliness is assessed using the 3-item UCLA loneliness scale for children [68] to consider participants' subjective loneliness. Items are summed to create a total score (3-12), whereby higher results indicate greater loneliness.

Engagement Measures

Engagement with Purrble will be assessed using two measures; a bespoke survey [59] and an adapted version of Twente Engagement with eHealth Technologies Scale (TWEETS) [69]. These will be deployed as part of the main assessment for participants allocated to the intervention group.

The bespoke survey [59] enquires about Purrble use and perceived usefulness over four items. These are analysed separately, with qualitative responses indicating contexts or situations where Purrble has been found helpful or unhelpful.

TWEETS [69] quantitatively measures intervention engagement across nine items. This is split into subsections considering behaviour, cognitive, and affective engagement. Total scores range from 0-36, with higher scores indicating greater engagement.

For peer review only

Supplementary material 4

STANDARD PROTOCOL ITEMS: RECOMMENDATIONS FOR INTERVENTIONAL TRIALS

SPIRIT 2013 Checklist: Recommended items to address in a clinical trial protocol and related documents*

Section/item	Item No	Description	Page and line No
**Administrative information**			
Title	1	Descriptive title identifying the study design, population, interventions, and, if applicable, trial acronym	Page 1 1-4
Trial registration	2a	Trial identifier and registry name. If not yet registered, name of intended registry	Page 2 44
	2b	All items from the World Health Organization Trial Registration Data Set	n.a.
Protocol version	3	Date and version identifier	n.a.
Funding	4	Sources and types of financial, material, and other support	Page 13 399-401
Roles and responsibilities	5a	Names, affiliations, and roles of protocol contributors	Page 1 5-13<br><br>Page 13 391-396
	5b	Name and contact information for the trial sponsor	Page 13 400-401
	5c	Role of study sponsor and funders, if any, in study design; collection, management, analysis, and interpretation of data; writing of the report; and the decision to submit the report for publication, including whether they will have ultimate authority over any of these activities	Page 13 401-402

For peer review only - http://bmjopen.bmj.com/site/about/guidelines.xhtml

	5d	Composition, roles, and responsibilities of the coordinating centre, steering committee, endpoint adjudication committee, data management team, and other individuals or groups overseeing the trial, if applicable (see Item 21a for data monitoring committee)	n.a.
**Introduction**			
Background and rationale	6a	Description of research question and justification for undertaking the trial, including summary of relevant studies (published and unpublished) examining benefits and harms for each intervention	Page 2-4<br>55-149
	6b	Explanation for choice of comparators	Page 6<br>195-199<br><br>Page 12<br>360-362
Objectives	7	Specific objectives or hypotheses	Page 4<br>150-161
Trial design	8	Description of trial design including type of trial (eg, parallel group, crossover, factorial, single group), allocation ratio, and framework (eg, superiority, equivalence, noninferiority, exploratory)	Page 4-5<br>163-178
**Methods: Participants, interventions, and outcomes**			
Study setting	9	Description of study settings (eg, community clinic, academic hospital) and list of countries where data will be collected. Reference to where list of study sites can be obtained	Page 6-7<br>204-222
Eligibility criteria	10	Inclusion and exclusion criteria for participants. If applicable, eligibility criteria for study centres and individuals who will perform the interventions (eg, surgeons, psychotherapists)	Page 6<br>201-209
Interventions	11a	Interventions for each group with sufficient detail to allow replication, including how and when they will be administered	Page 5-6<br>179-199
	11b	Criteria for discontinuing or modifying allocated interventions for a given trial participant (eg, drug dose change in response to harms, participant request, or improving/worsening disease)	Page 7<br>234-235

For peer review only

	11c	Strategies to improve adherence to intervention protocols, and any procedures for monitoring adherence (eg, drug tablet return, laboratory tests)	Page 8<br>285-287
	11d	Relevant concomitant care and interventions that are permitted or prohibited during the trial	n.a.
Outcomes	12	Primary, secondary, and other outcomes, including the specific measurement variable (eg, systolic blood pressure), analysis metric (eg, change from baseline, final value, time to event), method of aggregation (eg, median, proportion), and time point for each outcome. Explanation of the clinical relevance of chosen efficacy and harm outcomes is strongly recommended	Page 8-10<br>268-292<br><br>SupMat3
Participant timeline	13	Time schedule of enrolment, interventions (including any run-ins and washouts), assessments, and visits for participants. A schematic diagram is highly recommended (see Figure)	Page 6-7<br>211-244
Sample size	14	Estimated number of participants needed to achieve study objectives and how it was determined, including clinical and statistical assumptions supporting any sample size calculations	Page 7-8<br>245-267
Recruitment	15	Strategies for achieving adequate participant enrolment to reach target sample size	Page 6-7<br>211-222
**Methods: Assignment of interventions (for controlled trials)**			
Allocation:			
Sequence generation	16a	Method of generating the allocation sequence (eg, computer-generated random numbers), and list of any factors for stratification. To reduce predictability of a random sequence, details of any planned restriction (eg, blocking) should be provided in a separate document that is unavailable to those who enrol participants or assign interventions	Page 7<br>224-228
Allocation concealment mechanism	16b	Mechanism of implementing the allocation sequence (eg, central telephone; sequentially numbered, opaque, sealed envelopes), describing any steps to conceal the sequence until interventions are assigned	Page 7<br>224-228
Implementation	16c	Who will generate the allocation sequence, who will enrol participants, and who will assign participants to interventions	Page 7<br>224-231

Blinding (masking)	17a	Who will be blinded after assignment to interventions (eg, trial participants, care providers, outcome assessors, data analysts), and how	Page 7 224-231
	17b	If blinded, circumstances under which unblinding is permissible, and procedure for revealing a participant's allocated intervention during the trial	n.a.
**Methods: Data collection, management, and analysis**			
Data collection methods	18a	Plans for assessment and collection of outcome, baseline, and other trial data, including any related processes to promote data quality (eg, duplicate measurements, training of assessors) and a description of study instruments (eg, questionnaires, laboratory tests) along with their reliability and validity, if known. Reference to where data collection forms can be found, if not in the protocol	Page 4-5 163-178<br><br>Page 7-10 245-292<br><br>SupMat3
	18b	Plans to promote participant retention and complete follow-up, including list of any outcome data to be collected for participants who discontinue or deviate from intervention protocols	n.a.
Data management	19	Plans for data entry, coding, security, and storage, including any related processes to promote data quality (eg, double data entry; range checks for data values). Reference to where details of data management procedures can be found, if not in the protocol	Digital Youth management agreement
Statistical methods	20a	Statistical methods for analysing primary and secondary outcomes. Reference to where other details of the statistical analysis plan can be found, if not in the protocol	Page 11-12 307-357
	20b	Methods for any additional analyses (eg, subgroup and adjusted analyses)	Page 11-12 327-336<br><br>352-357
	20c	Definition of analysis population relating to protocol non-adherence (eg, as randomised analysis), and any statistical methods to handle missing data (eg, multiple imputation)	n.a.
**Methods: Monitoring**			

Data monitoring	21a	Composition of data monitoring committee (DMC); summary of its role and reporting structure; statement of whether it is independent from the sponsor and competing interests; and reference to where further details about its charter can be found, if not in the protocol. Alternatively, an explanation of why a DMC is not needed	Page 13 400-403
	21b	Description of any interim analyses and stopping guidelines, including who will have access to these interim results and make the final decision to terminate the trial	n.a.
Harms	22	Plans for collecting, assessing, reporting, and managing solicited and spontaneously reported adverse events and other unintended effects of trial interventions or trial conduct	Page 7 237-244<br><br>SupMat5
Auditing	23	Frequency and procedures for auditing trial conduct, if any, and whether the process will be independent from investigators and the sponsor	n.a.
**Ethics and dissemination**			
Research ethics approval	24	Plans for seeking research ethics committee/institutional review board (REC/IRB) approval	Page 12-13 367-369
Protocol amendments	25	Plans for communicating important protocol modifications (eg, changes to eligibility criteria, outcomes, analyses) to relevant parties (eg, investigators, REC/IRBs, trial participants, trial registries, journals, regulators)	Page 13 375-381
Consent or assent	26a	Who will obtain informed consent or assent from potential trial participants or authorised surrogates, and how (see Item 32)	Page 7 224-225<br><br>Page 12-13 369-372
	26b	Additional consent provisions for collection and use of participant data and biological specimens in ancillary studies, if applicable	n.a.
Confidentiality	27	How personal information about potential and enrolled participants will be collected, shared, and maintained in order to protect confidentiality before, during, and after the trial	SupMat2
Declaration of interests	28	Financial and other competing interests for principal investigators for the overall trial and each study site	Page 13-14 404-410

For peer review only

Access to data	29	Statement of who will have access to the final trial dataset, and disclosure of contractual agreements that limit such access for investigators	Page 14 412-415
Ancillary and post-trial care	30	Provisions, if any, for ancillary and post-trial care, and for compensation to those who suffer harm from trial participation	n.a.
Dissemination policy	31a	Plans for investigators and sponsor to communicate trial results to participants, healthcare professionals, the public, and other relevant groups (eg, via publication, reporting in results databases, or other data sharing arrangements), including any publication restrictions	Page 13 375-381
	31b	Authorship eligibility guidelines and any intended use of professional writers	n.a.
	31c	Plans, if any, for granting public access to the full protocol, participant-level dataset, and statistical code	n.a.
**Appendices**			
Informed consent materials	32	Model consent form and other related documentation given to participants and authorised surrogates	SupMat2
Biological specimens	33	Plans for collection, laboratory evaluation, and storage of biological specimens for genetic or molecular analysis in the current trial and for future use in ancillary studies, if applicable	n.a.

*It is strongly recommended that this checklist be read in conjunction with the SPIRIT 2013 Explanation & Elaboration for important clarification on the items. Amendments to the protocol should be tracked and dated. The SPIRIT checklist is copyrighted by the SPIRIT Group under the Creative Commons "Attribution-NonCommercial-NoDerivs 3.0 Unported" license.

For peer review only

Supplementary material 5

Safeguarding Procedures

The safeguarding procedures will be presented to potential participants in the information sheet before they consent to the study. These were codesigned and agreed with Sprouting Minds, based on previous studies conducted with young people with self-harmful experiences [1-3]. To support young people's safety during the study, the following measures will be instituted:

1. Before starting the study, all participants are required to attend an individual study briefing. At this point, they are taken through the study outline, invited to ask questions, and asked to create a safety plan [4] with the support of a specially trained researcher. Safety planning is quick to complete, is tangible to participants and has been shown to be effective at reducing self-harmful thoughts and behaviours [5]. Participants keep this safety plan containing their individualised strategies for their personal use beyond the life of the study. During the wellbeing check, participants are asked to reflect on their safety plan if they feel distressed at any other point during the study. This follows safety protocols used in other studies by the researcher [1-3].
2. During the briefing, participants will need to nominate a support contact (parent, friend over 18 years, GP, etc), with the understanding that if they do not respond to reactive safeguarding (which can be triggered for each of the main assessments), this person will be contacted to ensure that the participant is safe.
    a. This nominated person will be contacted (via email) to inform them that the participant is taking part in a mental health study and that we will reach out if we are unable to contact the participant following indication that they are at increased risk.
3. All assessments will include signposting to additional supports (e.g., Young Minds, Samaritans, Papyrus) as well as encouraging participants to seek help from their GP if they are distressed.
4. All assessments will include a visual scale to rate mood pre- and post- completing the survey (1 - very distressed - 10 extremely happy). This is to examine whether the assessment process has an impact, positively or negatively, on the participant. Such assessments have been successfully used in prior self-harm research by the research team members as an indicator of assessment impact [36].
5. Participants in need of support will be identified by checking their responses to the main battery of assessments, within 24 hours. If participants respond that they have had experiences of self-harmful or suicidal thoughts and self-harm behaviour, as well as showing a that the assessment has had a negative impact, they receive a wellbeing call the following day between 1pm-4pm. This allows enough time for the researcher to identify participant risk and inform the PI.
    a. If the participant does not pick up the phone, they will be contacted via email, asking about their wellbeing, whether they wish to continue with the study, and to arrange a time convenient to complete a wellbeing check. During the wellbeing check, the researcher will be empathic to the difficulties of the participant. The participant will be asked whether they wish to continue the study and, if so, they

For peer review only - http://bmjopen.bmj.com/site/about/guidelines.xhtml

will update their safety plan with the researcher. If participants do not wish to continue the study, they will be allowed to withdraw with no consequences. They will be invited to interview to discuss their thoughts, opinions and experiences of the study. This would also ask about why they withdrew from the study.

b. If a participant still does not respond to the email, the same procedure will be attempted twice more. If there is still no contact from the participant by day 4, their support contact (nominated during briefing) will be contacted.

Alongside this, all researchers who undertake briefing (including safety planning) and wellbeing calls will be invited to take part in group supervision sessions once a week during data collection with leading authors. This will be a confidential, safe space to discuss researchers' wellbeing and mental health, with support for any difficulties which may surface.

References

1. Williams AJ, Arcelus J, Townsend E, Michail M. Feasibility and acceptability of experience sampling among LGBTQ+ young people with self-harmful thoughts and behaviours. Frontiers in psychiatry. 2022 Aug 17;13:916164. Doi.org/10.3389/fpsyt.2022.916164
2. Williams AJ, Arcelus J, Townsend E, Michail M. Understanding the processes underlying self-harm ideation and behaviors within LGBTQ+ young people: A qualitative study. Archives of suicide research. 2021 Apr 3;27(2):380-96. doi.org/10.1080/13811118.2021.2003273
3. Williams, AJ, Townsend, T, Naeche, N, Chapman-Nisar, A, Hollis, C, Slovak, P. Investigating the feasibility, acceptability and appropriation of a socially assistive robot among minority youth at-risk of self-harm. [in prep]
4. Stanley B, Brown GK. Safety planning intervention: a brief intervention to mitigate suicide risk. Cognitive and behavioral practice. 2012 May 1;19(2):256-64. doi.org/10.1016/j.cbpra.2011.01.001
5. Nuij C, van Ballegooijen W, De Beurs D, Juniar D, Erlangsen A, Portzky G, O'Connor RC, Smit JH, Kerkhof A, Riper H. Safety planning-type interventions for suicide prevention: meta-analysis. The British Journal of Psychiatry. 2021 Aug;219(2):419-26. doi.org/10.1192/bjp.2021.50

For peer review only
